# Catalog of Differentially Expressed Long Non-Coding RNA following Activation of Human and Mouse Innate Immune Response

**DOI:** 10.3389/fimmu.2017.01038

**Published:** 2017-08-29

**Authors:** Benoit T. Roux, James A. Heward, Louise E. Donnelly, Simon W. Jones, Mark A. Lindsay

**Affiliations:** ^1^Department of Pharmacy and Pharmacology, University of Bath, Bath, United Kingdom; ^2^Barts Cancer Institute, Queen Mary University of London, London, United Kingdom; ^3^Airway Disease, National Heart and Lung Institute, Imperial College, London, United Kingdom; ^4^Institute of Inflammation and Ageing, MRC-ARUK Centre for Musculoskeletal Ageing Research, University of Birmingham, Birmingham, United Kingdom

**Keywords:** long non-coding RNA, innate immunity, conserved motif, human catalog, mouse catalog, inflammation

## Abstract

Despite increasing evidence to indicate that long non-coding RNAs (lncRNAs) are novel regulators of immunity, there has been no systematic attempt to identify and characterize the lncRNAs whose expression is changed following the induction of the innate immune response. To address this issue, we have employed next-generation sequencing data to determine the changes in the lncRNA profile in four human (monocytes, macrophages, epithelium, and chondrocytes) and four mouse cell types (RAW 264.7 macrophages, bone marrow-derived macrophages, peritoneal macrophages, and splenic dendritic cells) following exposure to the pro-inflammatory mediators, lipopolysaccharides (LPS), or interleukin-1β. We show differential expression of 204 human and 210 mouse lncRNAs, with positional analysis demonstrating correlation with immune-related genes. These lncRNAs are predominantly cell-type specific, composed of large regions of repeat sequences, and show poor evolutionary conservation. Comparison within the human and mouse sequences showed less than 1% sequence conservation, although we identified multiple conserved motifs. Of the 204 human lncRNAs, 21 overlapped with syntenic mouse lncRNAs, of which five were differentially expressed in both species. Among these syntenic lncRNA was *IL7-AS* (antisense), which was induced in multiple cell types and shown to regulate the production of the pro-inflammatory mediator interleukin-6 in both human and mouse cells. In summary, we have identified and characterized those lncRNAs that are differentially expressed following activation of the human and mouse innate immune responses and believe that these catalogs will provide the foundation for the future analysis of the role of lncRNAs in immune and inflammatory responses.

## Introduction

High-throughput sequencing indicates that much of the human genome is transcribed into non-coding RNAs (ncRNAs) with estimates of the proportion varying from ~62% predicted by the ENCODE project (1) to ~10% based on evolutionary conservation (2). By absolute amount, the majority of ncRNAs (>90%) are involved in house-keeping activities such as translation, splicing, and post-transcriptional RNA modifications and include ribosomal RNAs, transfer RNAs, short nucleolar RNAs, and small nuclear RNAs (3, 4). The remaining ncRNAs are broadly classified as either short ncRNAs [<200 nucleotides (nt)] or long ncRNAs (lncRNAs) (>200 nt) (4). The microRNA family of short ncRNAs is the best characterized and is known to induce messenger RNA (mRNA) degradation or block mRNA translation *via* the RNA interference pathway (5). By contrast, much less in known about lncRNAs, although, by comparison with mRNAs, their expression is cell specific and they are generally shorter in length, contain fewer exons, and are expressed at lower levels (6, 7). Presently, lncRNAs are classified by their relative position to protein-coding mRNAs and include the long intergenic ncRNAs (lincRNAs), antisense (AS), and pseudogenes (8). Although there is accumulating evidence showing that lncRNAs are regulators of a host of physiological and pathological responses, our understanding of their mechanism of action is limited. By analogy with protein-coding genes, it has been speculated that this is mediated through domains that interact with proteins and/or base pair with RNA/DNA (9). However, the identification of these domains has been hindered by their poor evolutionary conservation, which, in contrast to protein-coding genes, does not require the maintenance of a conserved open reading frame for optimal translation (6). Instead, it is thought that the lncRNAs conservation is geared toward the maintenance of genomic position (synteny), short domains (microdomains), and secondary structure (7, 10).

The innate immune response provides the initial defense against infection by external pathogens through induction of an inflammatory response. The presence of pathogens is commonly detected by cells of the myeloid family, including tissue resident macrophages, dendritic cells, and circulating blood monocytes (11, 12). These cells express families of pattern recognition receptors that bind conserved molecules within bacteria, fungi, and viruses including lipoproteins, lipopolysaccharides (LPS), bacterial CpG motifs, and single/double-stranded RNA. Many families of pattern recognition receptors have been identified, although the best characterized are the toll-like receptor and interleukin-1β (IL1β) receptor superfamily. Activation of Toll-like receptors stimulates the production of inflammatory mediators *via* transcription factors including nuclear factor-κB (NF-κB). This leads to a spectrum of responses including the release of multiple inflammatory mediators, as well as the activation of the inflammasome and the subsequent production of IL1β. The latter then induces a potent inflammatory response in the surrounding stromal cells such as the epithelium, chondrocytes, and fibroblasts (11, 12).

Recent publications have identified a number of lncRNAs that are differentially expressed following activation of innate immunity and which regulate the subsequent inflammatory response. In human cells, these include *PACER* (p50-associated COX-2 extragenic RNA) (13), *THRIL* (TNFα- and hnRNPL-related immunoregulatory lincRNA) (14), *lnc-IL7R* (15), and *IL1β-RBT46* (16), while studies in mice have identified *lincRNA-COX2* (17, 18), *lincRNA-EPS* (19), and *lincRNA-Tnfaip3* (20). However, despite these early indications that lncRNAs act as novel regulators, there has been no systematic attempt to identify lncRNAs whose expression is changed following the induction of the innate immune response. To address this issue, we determined the changes in lncRNA profile in four human and four mouse cell types following exposure to LPS or IL1β. From this analysis, we have cataloged and characterized 204 human and 210 mouse lncRNAs that are differentially expressed following activation of the innate immune response. We have then employed this list of potentially immune modulatory lncRNAs, to identify conserved microdomains and syntenic lncRNAs. To confirm the biological relevance of this analysis, we have shown that the lncRNA *IL7-AS* [located AS to interleukin-7 (IL7) gene] is induced across multiple human and mouse cell types and regulates the expression and hence the release of the pro-inflammatory mediator, interleukin-6 (IL6).

## Materials and Methods

### Isolation and Treatment of Human Monocytes and Macrophages

Human monocytes were prepared as previously described (16). To obtain monocyte-derived macrophages, monocytes were re-suspended in MDM complete media [RPMI-1640 supplemented with 10% (v:v) Fetal Calf Serum, 2 mM l-glutamine, 100 U/ml penicillin, and 100 µg/ml streptomycin; all GIBCO, Life Technologies] and seeded onto six-well black plates (10^6^ cells/well) for 2 h at 37°C, 5% (v:v) CO_2_ to allow monocytes to adhere to the plate. Non-adherent cells were aspirated, and monocytes were incubated with fresh complete media containing GM-CSF (2 ng/ml; R&D Systems). Monocytes were incubated at 37°C, 5% (v:v) CO_2_ for 12 days to allow full differentiation into MDMs; fresh media containing GM-CSF were replenished on days 4 and 7. Cells were treated with 10 ng/ml LPS for 4 h, and the controls were left untreated. The media was then removed, and the cells lysed prior to RNA extraction. Circulating blood was collected upon obtaining informed consent, and the study was approved by the National Research Ethics Service (NRES 13/LO/0354).

### Production and Treatment of Human Chondrocytes

For the isolation of primary human chondrocytes, articular cartilage was digested using filter-sterilized collagenase IIA (2 mg/ml; Sigma Aldrich) for 5 h at 37°C. Digested cartilage was then filtered by passing through a 40-µm cell strainer (BD Biosciences), and the filtrate centrifuged. Chondrocytes were then resuspended in growth media [DMEM supplemented with 10% (v:v) FCS, 2 mM l-glutamine, 100 U/ml penicillin, 100 µg/ml streptomycin, non-essential amino acids 5% (v:v); all GIBCO, Life Technologies, and 2 µg/ml amphotericin; Sigma Aldrich]. Cells were grown to 70–80% confluence and, then, either stimulated with 1 ng/ml IL1β for 4 h, and the controls were left untreated. OA patient joint tissue was collected from the Royal Orthopaedic Hospital (Birmingham) upon obtaining informed consent from patients undergoing elective joint replacement surgery. The study was approved by the NRES (14/ES/1044).

### Culture and Treatment of Human Epithelial A549 Cells

Human epithelial A549 cells were cultured in growth media [DMEM/F-12 supplemented with 10% (v:v) FCS, 2 mM l-glutamine, 100 U/ml penicillin, and 100 µg/ml streptomycin; all GIBCO, Life Technologies] and incubated in a 37°C, 5% (v:v) CO_2_ humidified incubator. For all experiments, A549 cells were seeded in 24-well plate at 1–5 × 10^5^ cell/well and stimulated with 30 ng/ml IL1β (recombinant, *Escherichia coli*; Sigma Aldrich) for 4 and 24 h, and the controls were left untreated.

### Culture and Treatment of Human Monocytic THP-1 Cells

THP-1 cells were cultured in growth media [RPMI supplemented with 10% (v:v) FCS, 2 mM l-glutamine, 100 U/ml penicillin, 100 µg/ml streptomycin, and 50 nM of 2-mercaptoethanol; all GIBCO, Life Technologies] and incubated in a 37°C, 5% (v:v) CO_2_ humidified incubator. For all experiments, THP-1 cells were seeded in 24-well plate at 5–8 × 10^5^ cell/well and stimulated with 1 µg/ml LPS (*E. coli 055:B5*; Sigma Aldrich) for 4 and 24 h, and the controls were left untreated.

### Culture and Treatment of Mouse RAW 264.7 Macrophages

RAW 264.7 cells were cultured in growth media [DMEM supplemented with 10% (v:v) FCS, 2 mM l-glutamine, 100 U/ml penicillin, and 100 µg/ml streptomycin; all GIBCO, Life Technologies] and incubated in a 37°C, 5% (v:v) CO_2_ humidified incubator. For all experiments, RAW cells were seeded in 24-well plate at 2–5 × 10^5^ cell/well and stimulated with 1 µg/ml LPS (*E. coli 055:B5*; Sigma Aldrich) for 4 and 24 h, and the controls were left untreated.

### RNA Isolation and Quality Control

For all samples, total RNA was extracted using the RNeasy kit (Qiagen), included an on-column DNase treatment (Qiagen), according to the manufacturer’s guideline. RNA concentration was determined using the Qubit 2.0 (Life Technologies). RNA quality was measured using the Agilent Bioanalyser and produced RIN values >8.0.

### RNA Library Preparation and Sequencing

Total RNA from epithelial A549 and RAW 264.7 cells were purified using polyA + fractionation (Illumina), while the monocytes, macrophages, and synovial chondrocytes were subjected to ribosome depletion (Ribo-Zero, Illumina). For all tissues, cDNA libraries were prepared using the Illumina TruSeq Stranded Total RNA kit. Samples were then subjected to 100 bp, paired-end sequencing upon an Illumina 2000 or 2500 sequencing machine (Wellcome Trust Sequencing Unit, University of Oxford). Quality scores across sequenced reads were assessed using FASTQC v0.9.2.^1^ All samples were of high quality with the average score (mean and median) at each base across reads in each sample *Q* > 35. Historical mouse sequencing data were download from Sequence Read Archive (SRA)^2^ using the following command in SRA tools: fastq-dump -I --split-files <file_name>. This included data on bone marrow-derived macrophages (BMDMs) (ribozero, paired-end, and non-stranded, *n* = 2) (19), peritoneal macrophages, and splenic dendritic cells (ribozero, paired-end, and non-stranded, *n* = 2) (21).

### Alignment and Assembly of Human and Mouse lncRNAs

Paired-end reads were aligned to the human reference genome (hg38) using TopHat2 (version 2.1.0) (22) or the mouse reference genome (mm10) using Hisat2 (version 2.0.4) (23) using the following command line options. Tophat2: tophat --library-type fr-firststrand <reference_genome.gtf> -1 <forward_strand.fa> -2 <reverse_strand.fa> -o <output.sam>. Hisat2: hisat2 -q --dta --rna-strandness FR –x <reference_genone.gtf> -1 <forward_strand.fa> -2 <reverse-strand file.fa> –S <output.sam>. Output SAM files were then sorted and converted to BAM files (samtools sort -@ 8 –o output.bam output.sam) and indexed (samtools index –b output.bam) in Samtools (24). The BAM output files for all control and LPS or IL1β samples were merged using Bamtools (25) to produce two files per cell type. All possible genes from these two BAM files were assembled *ab initio* using StringTie (26, 27) using the following command line options: stringtie <input.bam> -o assembled_genes.gtf -e –A gene_quantification.txt. The eight GTFs containing the genes from across the four cell types (both control and activated) were then combined using Cuffmerge v2.2.1.0 (which is part of the Cufflinks suite) (28) to produce a “total” GTF containing all possible genes and converted into a BED file. The single and multiple exon genes were separated using the information obtained in column 10 (block/exon), and those genes <200 nucleotides were removed using the information in column 11 (exon lengths). The resulting two BED files containing single exon and multi-exonic genes were compared with Gencode v23 (29) using BEDtools 2 (30) to identify known and novel lncRNAs. Potential protein-coding genes were identified using the coding potential calculator^3^ (31). The GTF containing novel single and multi-exonic lncRNAs was concatenated with the Gencode v23 catalog (29), to produce a “master” human GTF employed for gene quantification using CuffNorm, Stringtie, and CuffDiff. Parallel analysis of the expression of protein-coding genes and lncRNAs in mouse was undertaken using Gencode m12 (32).

### Principle Component Analysis and Hierarchical Clustering

The abundance of potential lncRNAs and Gencode v23 defined genes in individual samples was defined as the fragments per kilobase exon per million reads mapped (FPKM) and determined using CuffNorm v2.2.1.1 (part of the Cufflinks suite) (28). PCA and hierarchical clustering on Gencode v23 genes demonstrating an expression >1 FPKM were performed using Genesis (v1.7.7) (33). Data were log2 transformed following the addition of 1 FPKM. The threshold for reporting gene expression at FPKM > 1 is based upon the ability to validate sequencing data using qRT-PCR (34).

### Differential mRNA and lncRNA Expression

The differential expression of assembled lncRNAs and Gencode-annotated protein-coding genes was assessed with the geometric option (DESeq) in Cuffdiff v2.2.1.3 (part of the Cufflinks suite) (28) using a significance threshold of *q* < 0.05. The command line options were as follows: cuffdiff --FDR = 0.05 --min-alignment-count = 10 --library-norm-method = geometric --dispersion-method = pooled -u <reference_genome.gtf> <control_1.bam>, <control_x.bam>, <activated_1.bam> <activated_x.bam> -o <output_file_name>.

### Assembly of lncRNA Gene Sequences

A BED file containing all the transcripts for each lncRNAs was extracted from the “master” GTF files and the exons extracted using the Gene BED to Exon/Intron/Codon BED expander (at www.usegalaxy.org) (35). Overlapping exons (genomic coordinates) from each transcript were merged using Bedtools 2 (30), the relevant DNA sequences were extracted using Extract Genomic DNA (at www.usegalaxy.org) (35), and all exons sequences merged to produce a FASTA of the lncRNA gene sequences.

### Determination of Evolutionary Conservation

A BED file containing all the transcripts for each lncRNAs was extracted from the master GTF files and submitted into the Table Browser Tool on the UCSC genome browser for comparative genomics^4^ (36).

### Identification and Removal of Repeat Sequences

Repeat sequences were identified and removed from the assembled lncRNA sequences (FASTA) using the default options in Repeatmasker.^5^

### Identification of Conserved Microdomains

Potential motifs within the lncRNA genes were identified by submitting gene sequences (FASTA) into MEME-ChIP option on MEME-Suite (37). Identification of conserved sequences between lncRNAs was undertaken with BLAST+ (38) by input of the FASTA files as both query and subject using the following command line: blastn -query <query_file.fasta> -subject <subject_file.fasta> -task blastn -outfmt “6 qacc sacc sseq pident qlen length evalue”> output.txt. These were compared with random control sequences of comparable lengths and AT ratios generated in the Build Controls Section (random sequences) of RSAT^6^ (39), while random protein-coding sequences of comparable lengths were selected from the mRNA sequences downloaded *via* Biomart in Ensembl.^7^ Output from all these BLASTn analyses (FASTA) was then submitted to MEME-ChIP.

### Identification of Syntenic lncRNAs

To identify syntenic lncRNA in the human and mouse catalogs, we used the Liftover program^8^ (36) to identify the predicted position of the human lncRNAs (hg38) on the mouse genome (mm10) and then examined whether these overlapped with the assembled mouse lncRNAs using Bedtools 2 (30).

### ChIPseq Analysis

ChIPseq sequencing files (FASTQ) containing information on H3K27ac and H3K4me3 deposition and related input controls (*n* = 2 per condition), from control and LPS-stimulated human monocytes at 4 h (GSE85245) (40), were download from SRA (see text footnote 2) using SRA tools: fastq-dump <file_name>. Sequencing data were aligned to hg38 using Bowtie 2 (41): bowtie2 -q --very-fast <reference_genome.gtf> -U <file_name.fastq> -S <output_file.sam>. Output SAM files were then sorted and converted to BAM files (samtools sort -@ 8 –o output.bam output.sam), indexed (samtools index –b output.bam) in Samtools (24), and then converted to BigWig format using BamCoverage, which is part of the deepTools suite (42) using the following command line: bamCoverage -b <input_bam.bam> --normalizeUsingRPKM --binSize 30 --smoothLength 300 -p 10 --extendReads 200 –o <output_file.bw>. Significant ChIPseq peaks (*n* = 2 pre-condition and *q* = <0.1) were called with MACS2 (43) using the broadpeak options: macs2 callpeak –t <sample_1> <sample_2> -c <control_1> <control_2> -broad <output_files> -g hs. The intersection between ChIPseq data (broadpeak.bed) and mRNA and lncRNA was undertaken using the Join option in the Operate upon Genomic Intervals section of Galaxy (at www.usegalaxy.org) (35). Heatmaps of the data were generated using deepTools. Matrices containing summary scores around promoters were generated from the H3Kme3 and H3K27ac BigWig files using the following options in the computeMatrix tool: computeMatrix reference-point -S <filename.bw> -R <mRNA/lncRNA.bed> -b 3000 -a 3000 -out <matrix.name>. Heatmaps were then generated using the plotHeatmap function and the following options: plotHeatmap -m <matrix.name> --colorMap YlOrRd --samplesLabel “<Sample Names>” -out <plot_name.eps>.

### Nuclear-Cytoplasm RNA Fractionation

A549 and THP-1 cells were stimulated with IL1β (30 ng/ml) or LPS (1 µg/ml) for 4 h respectively. The cells were scraped (i.e., A549) and/or collected, then centrifuged (12,000 × *g*, 1 min, 4°C). Supernatants were discarded, and the pellets resuspended in 1 ml of cold PBS and then split into two equal fractions (500 µl each). Fractions were then centrifuged, and their supernatants discarded. One of the two fractions was resuspended and lysed in 350 µl of RLT buffer (Qiagen) and constituted the whole lysate fraction. The pellet of the remaining fraction was resuspended in 175 µl of cold RLN buffer [50 mM Tris pH 8, 140 mM NaCl, 1.5 mM MgCl_2_, 0.5% (v:v) Non-idet P-40, 0.5 mM DDT, 1× Halt protease inhibitor cocktail; Thermo Fisher, and 20 U/ml SUPERase-IN; Ambion] and incubated on ice for ~15 min, in order to lyse the plasma membrane while leaving the nuclei intact. The nuclei were then isolated by centrifugation (300 × *g*, 10 min, 4°C). The supernatant was delicately collected and transferred into a fresh tube, and 600 µl of RLT buffer was added to it to constitute the cytoplasmic fraction while, the pellet was resuspended in 350 µl of RLT buffer to form the nuclear fraction. Total RNA was then extracted, and cDNA libraries were made using a set volume of RNA determined by the whole lysate fraction RNA quantity, within individual experiments. mRNA and RNA expressions were then determined using qPCR, and results were expressed as fold change compared to whole lysate stimulated samples.

### Transfection of THP-1, RAW 264.7, and A549 Cells with AS Locked Nucleic Acid GapmeRs

The following protocol was used to transfect all cell types (i.e., human monocytic THP-1 cells, mouse macrophage RAW 264.7 cells, and human epithelial A549 cells). AS Locked Nucleic Acid (LNA) GapmeRs (Exiqon, sequences are listed in Table S1 in Supplementary Material, final concentration of 30 nM) were mixed in 100 µl of serum- and antibiotic-free medium, supplemented with 5 µl of HiPerFect (Qiagen). Each mix was then added to each well of a 24-well plate. Cells were resuspended at desired concentration (1–8 × 10^5^ cells/well) in 100 µl of their corresponding complete medium and added on top of the LNA GapmeRs mixes. Cells were then incubated for 16 h. The following day cells were diluted with 400 µl of complete medium and stimulated with either LPS or IL1β. Cells’ supernatants were then collected for the analysis of cytokine release (see ELISA), and cells were lysed for RNA extraction at 4 and 24 h. mRNA and RNA expressions were then determined using qPCR, and results were expressed as fold change compared to non-transfected stimulated samples.

### Cytokine Release Measurement by ELISA

Following stimulation of A549, THP-1, and RAW 264.7 cells, supernatants were collected and measurements of human and mouse IL6 were made by ELISA (R&D Systems) according to the manufacturer’s guidelines. Results are expressed in percentage of maximum response of control stimulation.

### Availability of Data and Materials

All software, including the web address of the source code, is listed in Table S2 in Supplementary Material. The sequencing data are available from the gene expression omnibus under the following entries: human monocytes (ERA294222), human macrophages (GSE101868), human chondrocytes (GSE74220), human lung epithelial A549 cells (GSE101868), mouse RAW 264.7 macrophages (GSE101868), BMDMs (PRJEB11889), peritoneal macrophages, and splenic dendritic cells (SRP038980).

## Results

### Differential Expression of Protein-Coding Genes following Activation of the Human Innate Immune Response

**Figure 1:**
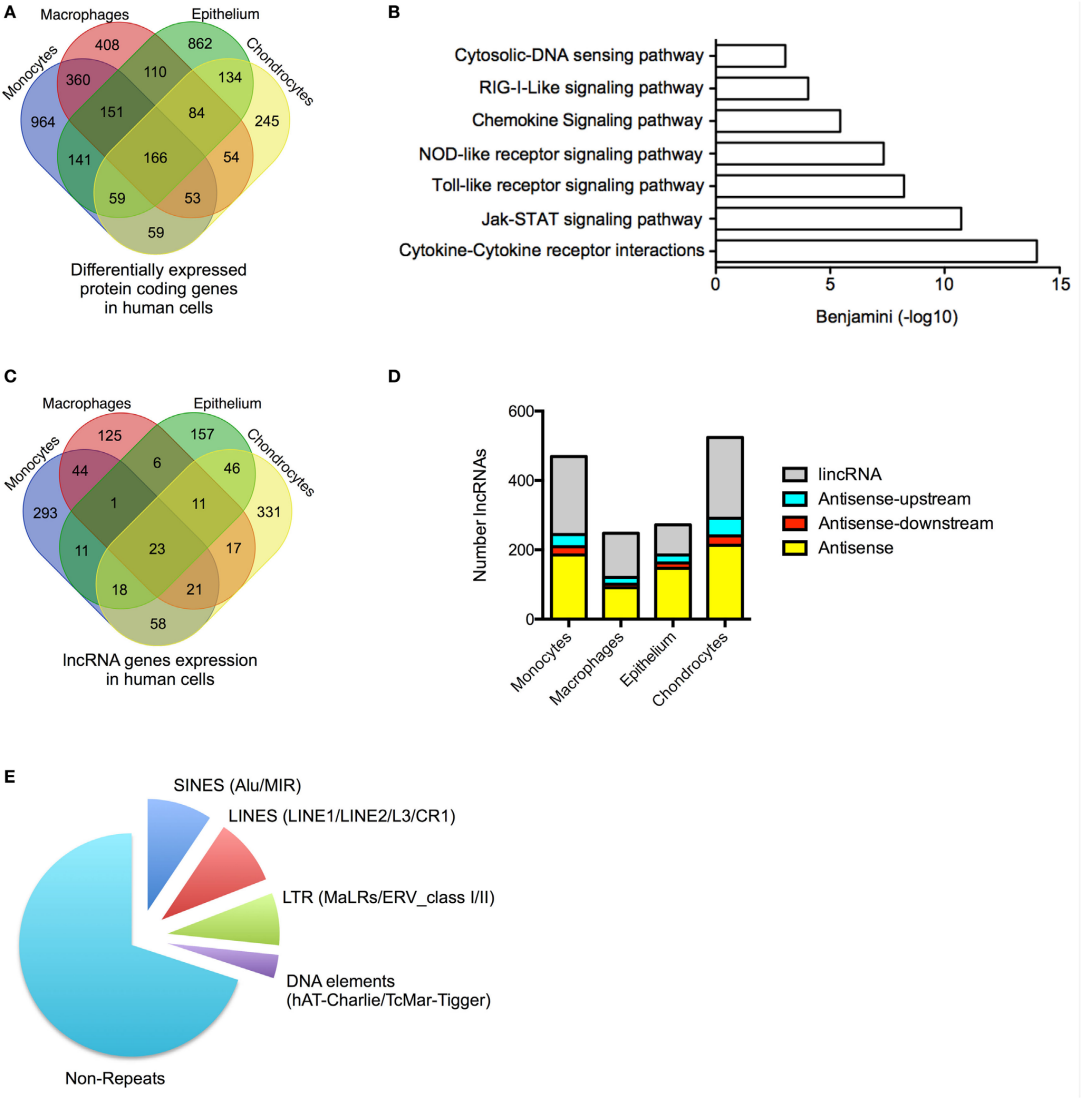
Profile of messenger RNAs’ (mRNAs) and long non-coding RNAs’ (lncRNAs) expressions in human cells. **(A)** Venn diagram showing the overlap in the differentially expressed mRNAs following lipopolysaccharides (LPS)-induced activation of monocytes and macrophages or IL1β-induced activation of epithelial cells and chondrocytes at 4 h. **(B)** Pathways analysis of the mRNAs that were differentially expressed across all human cell types. **(C)** Venn diagram showing the overlap in the lncRNAs expression profile in resting monocytes, macrophages, epithelial cells, and chondrocytes. **(D)** Distribution of different antisense and lincRNA species in resting monocytes, macrophages, epithelial cells, and chondrocytes and **(E)** Pie chart showing the percentage distribution of repeat sequences in the total lncRNA population obtained from all cell types.

We undertook stranded and paired-end sequencing data on total RNA obtained from four activated human cell types associated with the innate immune response including two myeloid immune cells, monocytes (ERA294222) and monocyte-derived macrophages (macrophages) (GSE101868), and two stromal cell types, lung A549 epithelial cells (GSE101868) and synovial chondrocytes (GSE74220). The myeloid cells were activated with bacterial LPS (*via* TLR4), while the stromal cells were activated using the pro-inflammatory cytokine, IL1β. Sequencing produced 2.0 billion reads (100 bases per read) of which 87% could be aligned to the human reference genome. Principle component analysis and unsupervised hierarchical clustering of the mRNA expression data (>1 FPKM) demonstrated separation of control and activated monocytes, macrophages, chondrocytes, and epithelial cells (Data Sheet S1 in Supplementary Material). Using the Gencode database (v23), we showed differential expression (*q* < 0.05) of 1,955 mRNAs in monocytes, 1,386 mRNAs in macrophages, 1,708 mRNAs in epithelial cells, and 855 mRNAs in chondrocytes (Table S3 in Supplementary Material). Integration of the data identified 3,853 mRNAs that were differentially expressed across all cell types. Of these, 2,479 (65%) were expressed in a single cell type, 858 (22%) were expressed in two cell types, 347 (9%) were expressed in three cell types, and 166 (4%) were expressed in four cell types (Figure 1A). As might be expected, KEGG pathway analysis (using DAVID bioinformatics platform) (44) showed that the 2,359 differentially up-regulated mRNAs across all four cell types were associated with activation of the innate immune response (Figure 1B). In contrast, the 1,494 down-regulated mRNAs were not associated with any pathways. These data indicated activation of the innate immune response in all four cell types following exposure to either of the pro-inflammatory mediators, LPS or IL1β.

### Profile of lncRNA Expression in Resting Human Cells

We identified 1,162 lncRNA genes that contained at least two exons and were expressed at >1 FPKM in at least one cell type (either control or stimulated cells) (Figure 1C; Table S4 in Supplementary Material). For clarity, we have included the “h” and “m” prefixes to identify human and mouse lncRNAs. Of these assembled genes, 586 overlapped with lncRNAs annotated in Gencode v23, meaning that the remaining 576 (50%) likely represent novel lncRNAs. Detailed breakdown identified 469 lncRNAs (54% novel) in monocytes, 248 lncRNAs (40% novel) in macrophages, 273 lncRNAs (32% novel) in epithelium, and 526 lncRNAs (44% novel) in chondrocytes. As in previous reports, we divided lncRNAs into four groups based upon their relative position to protein-coding genes: AS (overlapping a protein-coding gene on the opposite strand), AS-upstream (within 5 kb and located upstream/opposite strand from of a protein-coding genes), AS-downstream (within 5 kb and located downstream/opposite strand from of a protein-coding genes), and lincRNAs (located >5 kb from a protein-coding gene) (16). We have excluded lncRNAs located on the same strand and within 5 kb of a protein-coding gene, since these could potentially represent gene extensions. Using these criteria, it was found that lncRNAs could be subdivided into 39% AS, 5% AS-downstream, 8% AS-upstream, and 48% lincRNA, and this ratio remained similar across the four cell types (Figure 1D).

Examination of the overlap showed that the vast majority of lncRNA were expressed in a cell-specific manner with 906 (78%) selectively expressed in a single cell type, 182 (16%) in two cell types, 51 (4%) in three cell types, and 23 (2%) in all four cell types (Figure 1C; Table S4 in Supplementary Material). As previously reported (45), the lncRNAs were found to be enriched with repeat sequences (identified using repeatmasker.org) including 9.4% short interspersed nuclear elements (SINES), 9.7% long interspersed nuclear elements (LINES), 7.6% long terminal repeats (LTRs), and 3.4% DNA elements, leaving 70% of non-repeat sequence (Figure 1E).

We have identified 1,162 lncRNAs across the four human cell types including 576 novel lncRNAs that were enriched in repeat sequences and expressed in a predominantly cell-specific manner.

### Widespread Differential Expression of lncRNAs following Activation of the Human Innate Immune Response

**Figure 2:**
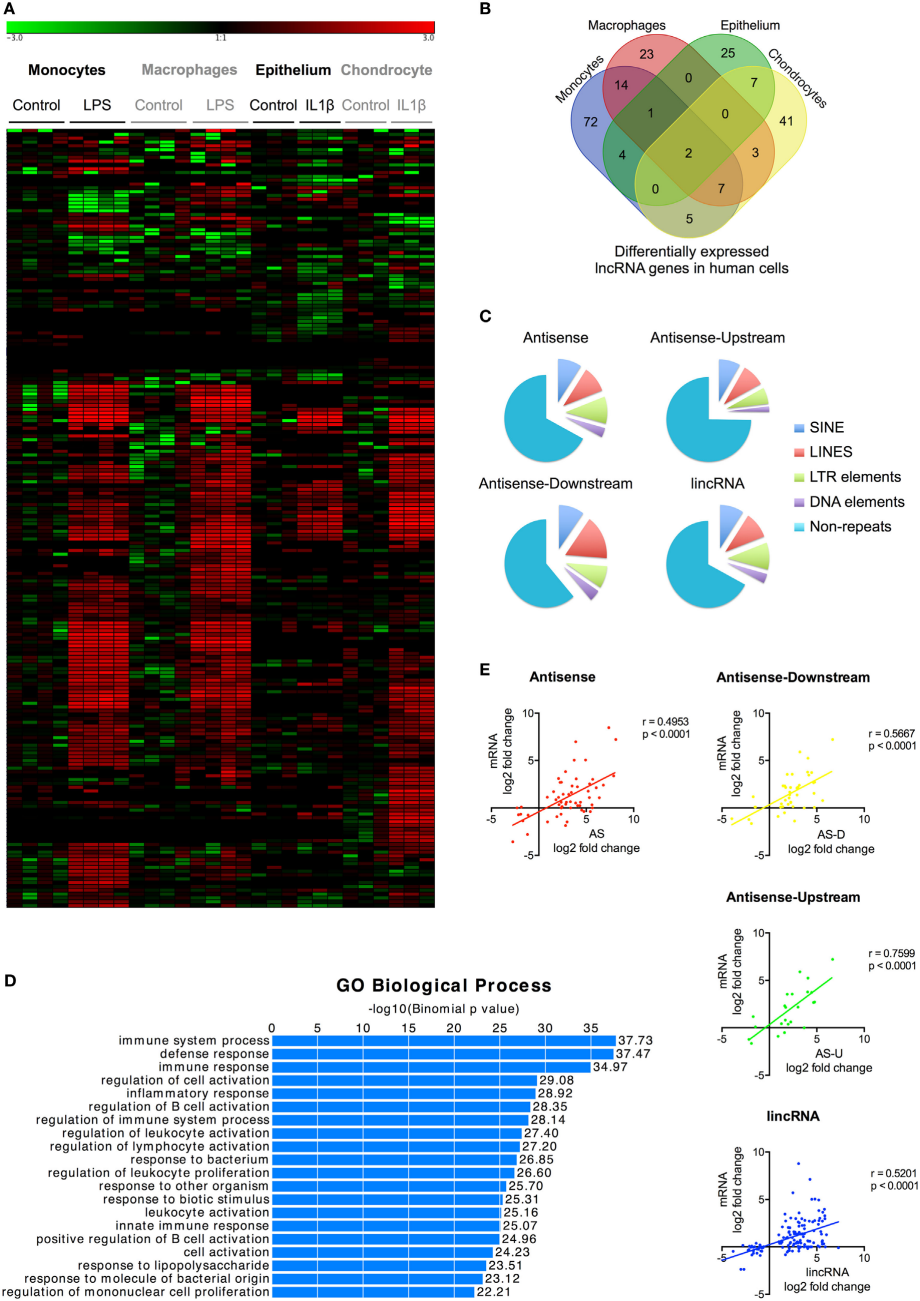
Characterization of differentially expressed long non-coding RNAs (lncRNAs) following activation of the human innate immune response. **(A)** Heatmap of the lncRNA expression levels in control and lipopolysaccharides (LPS) or interleukin-1β (IL1β) stimulated monocytes, macrophages, epithelium, and chondrocytes that have been subjected to unsupervised hierarchical based by experiment. **(B)** Venn diagram showing the overlap in the differentially expressed lncRNAs in LPS-stimulated monocytes and macrophages and IL1β-stimulated epithelial cells and chondrocytes at 4 h. **(C)** Pie charts showing the percentage distribution of repeat sequences in the various sub-populations of the differential expression lncRNAs across all four cell types with SINES = short interspersed nuclear elements, LINES = long interspersed nuclear elements, and LTR = long terminal repeat. **(D)** Pathways analysis of the messenger RNAs (mRNAs) located within 1 Mb of the differentially expressed lncRNA. **(E)** Pearson’s correlation between the differential expression of various lncRNA populations and that of the nearest mRNA.

To identify lncRNAs that might regulate the innate immune response, we examined their differential expression following exposure to either LPS (monocytes and macrophages) or IL1β (epithelium and chondrocytes) (Table S5 in Supplementary Material). We showed differential expression of l05 lncRNAs in monocytes, 50 lncRNAs in macrophages, 39 lncRNAs in epithelium, and 65 lncRNAs in chondrocytes (Figures 2A,B; Table S5 in Supplementary Material). This produced a total of 204 differentially expressed lncRNAs, which could be subdivided into 127 lincRNAs (62%), 45 AS (22%), 17 AS-downstream (8%), and 15 AS-upstream (8%). Comparison with Gencode v23 showed that 93 lncRNAs overlapped with annotated genes, indicating that the remaining 111 might be novel (Table S5 in Supplementary Material). Evaluation of the absolute change in expression across the four cell types showed a 10-fold difference between differentially expressed mRNAs and lncRNAs, with a mean (±SEM) of 55.5 ± 3.1 FPKM and 5.4 ± 3.1 FPKM, respectively. As with mRNAs, examination of the overlap between cell types showed that the vast majority (161 or 79%) were differentially expressed in a cell-specific manner (Figure 2B). Of the remainder, 33 (16%) were found in two cell types, eight (4%) in three cell types, and only two (1%) in all four cell types (Figure 2B).

Once again, large regions of repetitive sequences were found in AS (33%), AS-downstream (39%), AS-upstream (25%), and lincRNA (33%) (Figure 2C). To assess their potential function, we identified 699 genes located within 1 Mb of these differentially expressed lncRNAs using GREAT^9^ and showed that these were associated with immune activation and response (Figure 2D). Comparison of the fold change showed a correlation between expression of the nearest mRNA expression and that of the AS (*r* = 0.495, *p* < 0.0001), AS-downstream (*r* = 0.567, *p* < 0.0001), AS-upstream (*r* = 0.760, *p* < 0.0001), and lincRNAs (*r* = 0.520, *p* < 0.0001) (Figure 2E), which was not seen when we looked at the total lncRNA population (Data Sheet S2 in Supplementary Material).

In summary, we identified 204 lncRNAs that were differentially expressed across the four human cell types (including 111 novel lncRNAs) that could be subdivided into 62% lincRNAs, 22% AS, and 8% AS-downstream and 8% AS-upstream. The majority (161 lncRNAs) were expressed in a cell-specific manner, although there were 43 lncRNAs that were induced in multiple cell types. Positional analysis showed that lncRNA expression was correlated with immune-related genes and suggested that these might be functionally linked.

### Differential Expression of Single Exon lncRNAs during Activation of the Human Innate Immune Response

Although we had included only multi-exonic genes in our initial analysis, a number of the previous publications have identified single exon lncRNAs that regulate the innate response, including *PACER* (13) and *THRIL* (14). To provide a complete picture of the role of non-coding RNAs in the innate immune response, we therefore decided to include these in the analysis. Our *ab initio* assembly identified 44,656 single exon lncRNAs genes that were >200 nt and expressed at >1 FPKM in at least one cell type (either control or stimulated cells) (Table S6 in Supplementary Material). Significantly, the vast majority (42,085 or 94%) showed no overlap with annotated lncRNAs in Gencode v23, while breakdown by cell type identified 8,068 lncRNAs in monocytes, 313 lncRNAs in macrophages, 2,657 lncRNAs in epithelium, and 37,829 lncRNAs in chondrocytes. The wide variation between cell types indicated that identification might be influenced by sequencing variability and that many of these single exons lncRNAs likely represent artifacts.

**Figure 3:**
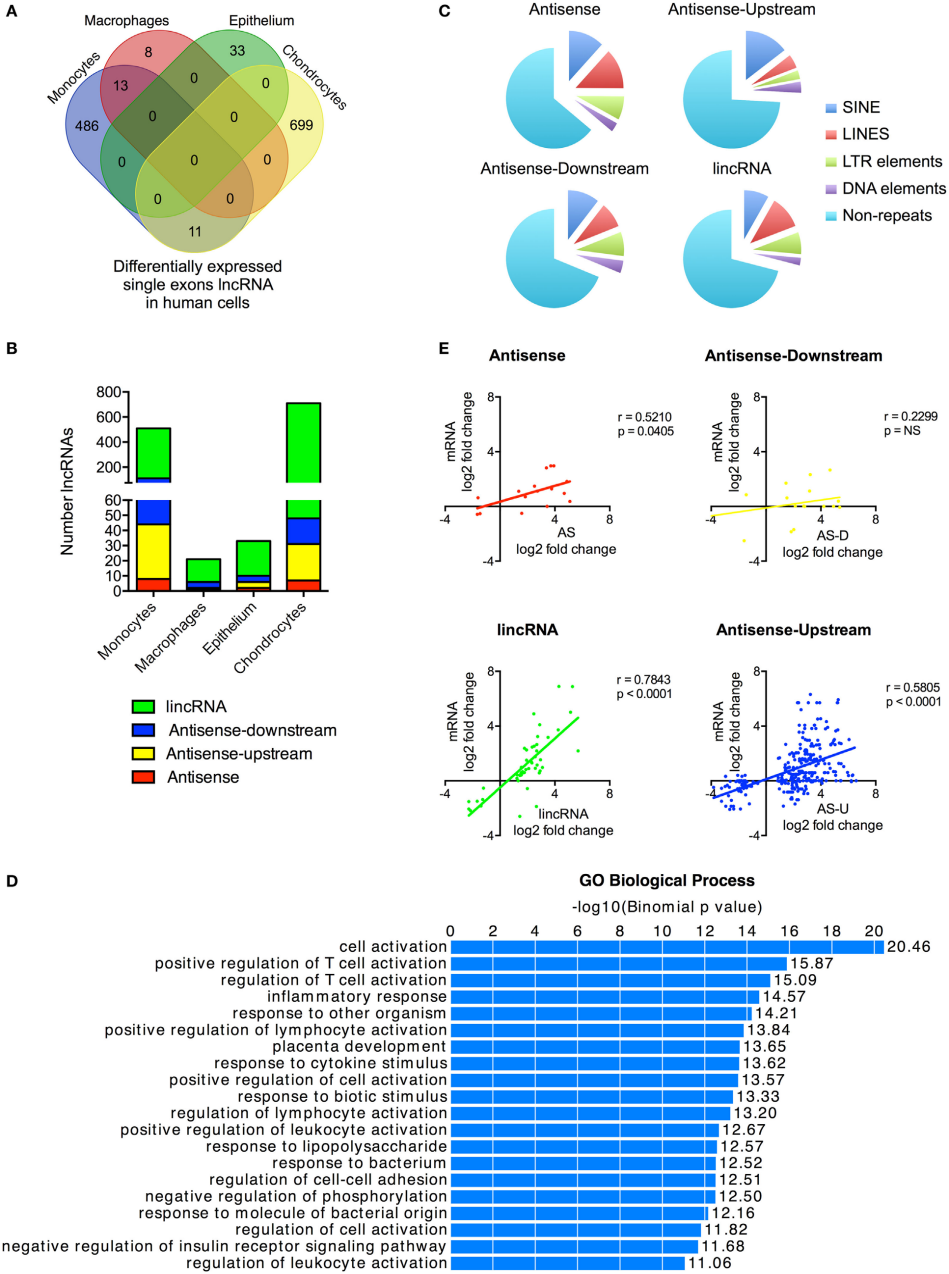
Characterization of differentially expressed single exon long non-coding RNAs (lncRNAs) following activation of the human innate immune response. **(A)** Venn diagram showing the overlap in the single exon lncRNAs expression in resting monocytes, macrophages, epithelial cells, and chondrocytes. **(B)** Distribution of different lncRNA species in resting monocytes, macrophages, epithelial cells, and chondrocytes, and **(C)** Pie chart showing the percentage distribution of repeat sequences in the various sub-populations of lncRNAs across all four cell types with SINES = short interspersed nuclear elements, LINES = long interspersed nuclear elements, and LTR = long terminal repeat. **(D)** Pathways’ analysis of the messenger RNAs (mRNAs) located within 1 Mb of the differentially expressed lncRNA. **(E)** Pearson’s correlation between the differential expression of various lncRNA populations and that of the nearest mRNA.

With these reservations in mind, we proceeded to identify those that were differentially expressed following activation of the innate immune response. Once again there was wide variation between cells with 510, 216, 33, and 710 differentially expressed lncRNAs in monocytes, macrophages, epithelium, and chondrocytes, respectively (Figure 3A). This produced a combined total of 1,250 lncRNAs across all four cell types, of which only a small proportion (3.5%) were shown to overlap with annotated lncRNAs in Gencode v23. Significantly, unlike the multi-exonic lncRNAs, only 24 of the 1,250 differentially expressed single exon lncRNAs were identified in two cell types and none were expressed in three or four cell types.

Using the percentage distribution across individual cell types, these were shown to be predominantly lincRNAs (78%), with much smaller numbers of AS (3%), AS-downstream (7%), and AS-upstream (12%) (Figure 3B). As might be expected, these single exon lncRNAs were shorter in length than the multi-exonic lncRNAs at 0.6 kb (1.9 kb; *p* < 0.0001), 1.0 kb (1.5 kb; *p* = 0.0039), and 2.1 kb (2.4 kb; *p* < 0.0001) for AS-downstream, AS-upstream, and lincRNA, respectively (numbers in brackets show the length for multi-exonic lncRNAs; paired statistical testing using Mann Whitney). The exception was the AS at 3.6 kb, which were longer than the 2.9 kb seen in multi-exonic AS (*p* < 0.0001). Comparison of the absolute change in expression gave a value of 3.0 ± 0.3 FPKM, a value not significantly different from 5.4 ± 3.1 FPKM seen with multi-exonic lncRNAs (Kruskal–Wallis test).

Once again, these single exon lncRNAs were also composed of large regions of repetitive sequences, which comprised 36, 31, 26, and 29% of the AS, AS-downstream, AS-upstream, and lincRNA sequences, respectively (Figure 3C). Assessment of their potential function identified 2,256 genes located within 1 Mb of these differentially expressed single exon lncRNAs and showed that these were also associated with immune activation and response (Figure 3D) and with the exception of AS-downstream that changes in expression correlated with those of the nearest mRNA (Figure 3E).

We were able to identify large numbers of differentially expressed single exon lncRNAs, whose expression was strongly cell type specific and correlated with that of adjacent immune-related genes. However, we speculate that the vast majority represent artifacts related to the computational analysis and/or local non-specific transcriptional activity. Interestingly, although we showed significant increases in the expression of PACER (*hXLOC_015084*) in monocytes and chondrocytes (Table S6 in Supplementary Material), we were unable to detect the presence of *THRIL* (14). This is purported to be embedded (in the AS direction) within the 3′-untranslated region (UTR) of *BRI3BP* but detailed visual inspection in monocytes and macrophages (as well as the other two cell types) failed to identify the presence of this lncRNA (Data Sheet S3 in Supplementary Material).

### LncRNA Expression in Human Monocytes Correlates with Activating Histone Marks

**Figure 4:**
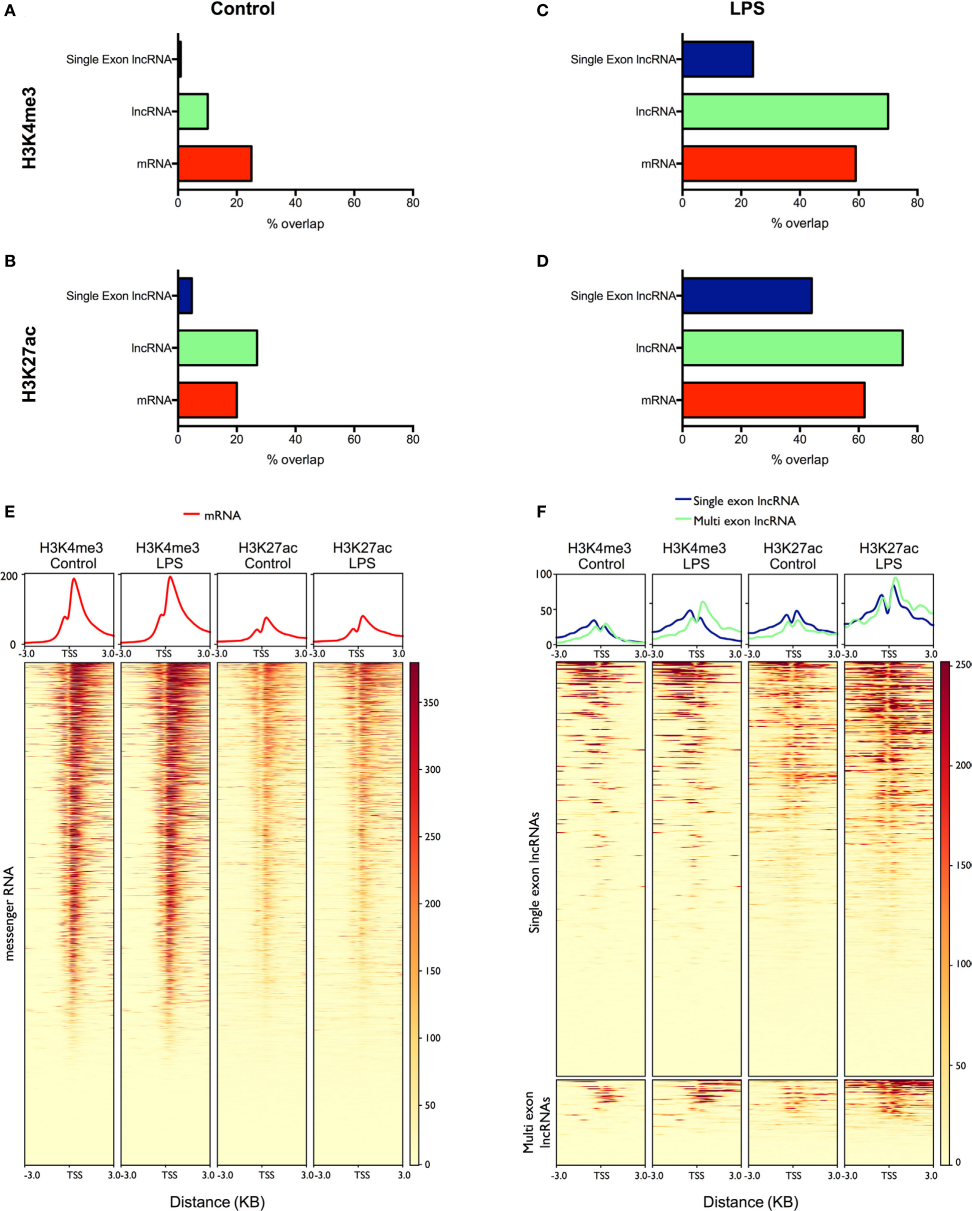
Analysis of the overlap between mRNAs/lncRNAs and histone marks in human monocytes ChIPSeq data from resting **(A,B)** and lipopolysaccharides (LPS)-stimulated **(C,D)** is expressed as the percentage of long non-coding RNA (lncRNA) and messenger RNA (mRNA) genes that overlap with peaks identified by MACS2 (% genomic overlap) for H3K4me3 (a marker of transcriptional activity) **(A,C)** and H3K27ac (a marker of active enhancers/promoters) **(B,D)**. Heatmaps were generated (lower panel) displaying the deposition of H3K4me3 and H3K27ac across the promoters (TSS ± 3 kb) of differentially expressed mRNAs **(E)** and lncRNAs **(F)**. The upper panel displays the mean deposition of reads across all of the regions in the heatmap.

To validate our lncRNA catalog, we examined in control and LPS-stimulated monocytes the overlap between the lncRNAs and two active histone marks; H3K4me3, a marker of transcriptional activity, and H3K27ac, a marker of active promoters and enhancers (40). Intersection of the peaks identified by MACS2 and the mRNAs expressed in resting monocytes showed a partial overlap with H3K4me3 (25%) (Figure 4A) and H3K27ac (20%) (Figure 4B). In comparison, the overlap between the multi-exonic lncRNAs and H3K4me3 was reduced at 10%, while there was an increased intersection with the deposition of H3K27ac at 27%. By contrast, there was little overlap between the single exon lncRNAs and H3K4me3 (1%) or H3K27ac (4%).

To examine the differentially expressed lncRNAs, we subsequently focused on the peaks identified following the same length of LPS stimulation in monocytes (4 h). As might be expected, the overlap between mRNAs and H3K4me3 (59%) (Figure 4C) and H3K27ac (62%) (Figure 4D) was greatly increased compared with resting cells and was comparable to the intersection seen with lncRNAs (H3K4me3 70% and H3K27ac 75%). In the case of differentially expressed single exons lncRNAs, the overlap was increased compared to controls (H3K4me3 24% and H3K27ac 44%) but did not reach the levels in mRNAs and lncRNAs. We further examined the profile of the two marks across the promoters (±3 kb) of the differentially expressed mRNAs and lncRNAs (Figures 4E,F). Although we were unable to detect a global increase in H3K4me3 and H3K27ac at the promoters of mRNAs (Figure 4E), the deposition of both H3K4me3 and H3K27ac was clearly increased for both the lncRNAs and single-exonic lncRNAs (Figure 4F). Overall, this ChIPseq analysis provides additional evidence to support our transcriptional analysis showing LPS-induced expression of multi-exonic lncRNAs and, to a lesser extent, single exon lncRNAs, in human monocytes. This also supports the existence of lncRNAs in resting monocytes, although the poor overlap with single exon lncRNAs indicates that many are indeed artifacts.

### Widespread Differential Expression of lncRNAs following Activation of the Mouse Innate Immune Response

**Figure 5:**
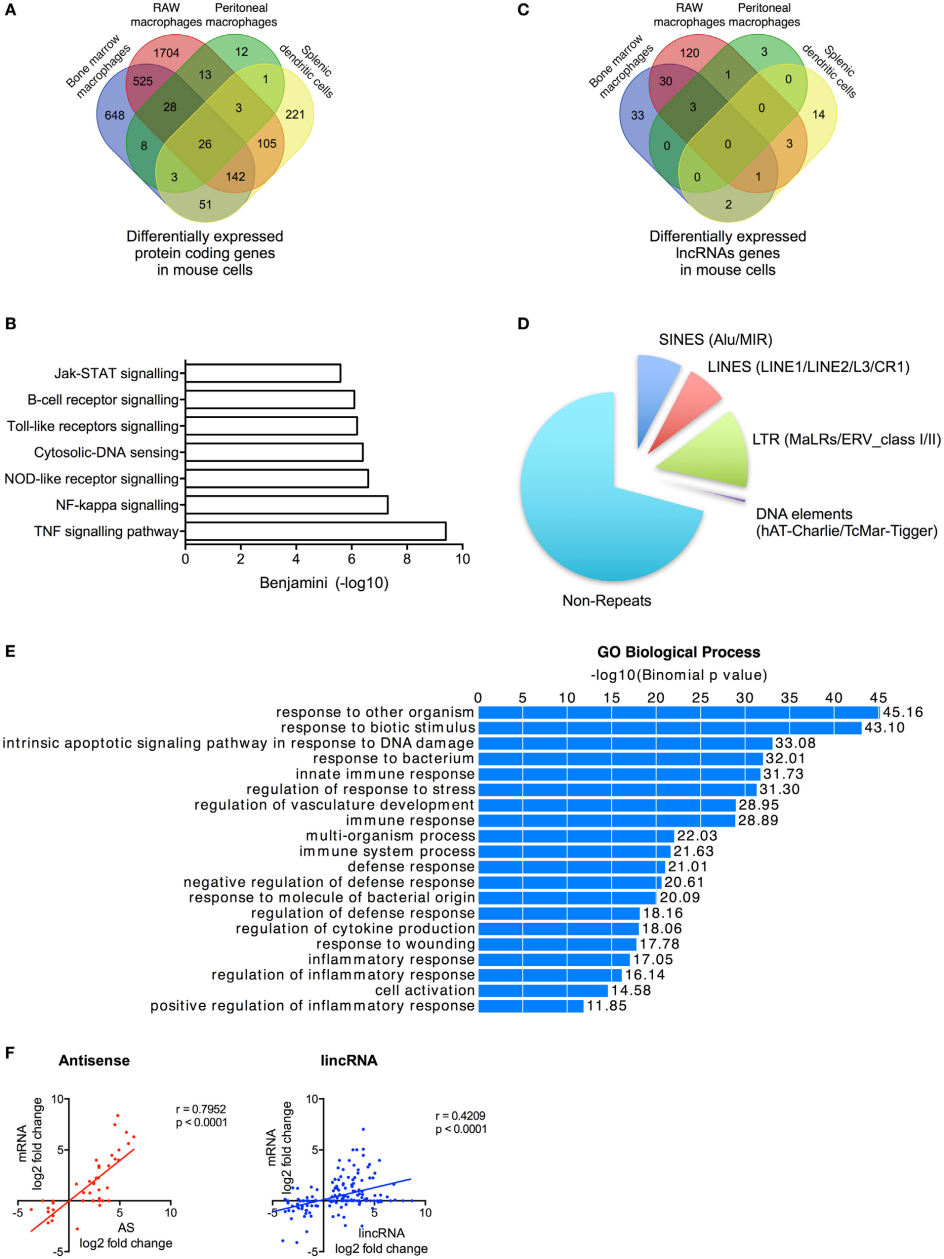
Characterization of differentially expressed long non-coding RNAs (lncRNAs) following activation of the mouse innate immune response. **(A)** Venn diagram showing the overlap in the differentially expressed messenger RNAs (mRNAs) in lipopolysaccharides (LPS)-stimulated bone marrow macrophages, RAW 264.7 macrophages, peritoneal macrophages, and splenic dendritic cells. **(B)** Pathway analysis of the differentially expressed mRNA across all cell types. **(C)** Venn diagram showing the overlap in the differentially expressed lncRNAs in LPS-stimulated bone marrow macrophages, RAW 264.7 macrophages, peritoneal macrophages, and splenic dendritic cells. **(D)** Pie charts showing the percentage distribution of repeat sequences in the differentially expressed lncRNAs across all four cell types with SINES = short interspersed nuclear elements, LINES = long interspersed nuclear elements, and LTR = long terminal repeat. **(E)** Pathways analysis of the mRNAs located within 1 Mb of the differentially expressed lncRNAs. **(F)** Pearson’s correlation between the differential expression of various lncRNA populations and that of the nearest mRNA.

Further studies were undertaken to identify and characterize the differentially expressed lncRNAs following activation of the mouse innate immune response and to compare these with human lncRNAs. To this end, we undertook sequencing of LPS-stimulated mouse RAW 264.7 macrophages and combined this with published sequencing data obtained from LPS-stimulated BMDMs (19), LPS-stimulated peritoneal macrophages (21), and LPS-stimulated splenic dendritic cells (21). Using the mouse Gencode database (m12), we showed differential expression (*q* < 0.05) of 1,293 mRNAs in BMDMs, 1,487 in RAW 264.7 macrophages, 90 in peritoneal macrophages, and 24 in dendritic cells (Figure 5A; Table S7 in Supplementary Material). As with the human cells, KEGG pathway analysis (using DAVID bioinformatics platform) (44) indicated activation of the immune response in all four cell types following exposure to LPS (Figure 5B).

Following *ab initio* assembly, we identified 2,386 lncRNAs across the four mouse cell types that could be divided into 869 lincRNAs (36%), 1,201 AS (50%), 170 AS-downstream (7%), and 146 AS-upstream (6%) (Table S8 in Supplementary Material). As a possible consequence of the poorer annotation of the mouse transcriptome, 1,592 (67%) of these were found to be novel lncRNAs. Following LPS stimulation, we showed differential expression of 210 lncRNAs (133 lincRNA, 34 AS, 19 AS-downstream, and 24 AS-upstream; *q* < 0.05) including 69 in BMDMs, 158 in RAW macrophages, 7 in peritoneal macrophages, and 20 in dendritic cells (Figure 5C; Table S8 in Supplementary Material). As with human cells, examination of the overlap between cell types showed that the vast majority (171 or 81%) were expressed in a cell-specific manner. Of the remainder, 36 were found in two cell types and 4 were found in three cell types (Figure 5C). Interestingly, our *ab initio* assembly identified three lncRNAs that have previously been shown to regulate the innate immune response: *lincRNA-COX2 (mXLOC_001674)* (17, 18), *lincRNA-EPS* (*mXLOC_029096*) (19), and *lincRNA-Tnfaip3 (mXLOC_003831)* (20). Differential expression in response to LPS was seen with *lincRNA-COX2* (BMDMs, RAW macrophages, and peritoneal macrophages) and *lincRNA-EPS* (RAW macrophages) but not *lincRNA-Tnfaip3* (Table S9 in Supplementary Material).

Characterization of these differentially expressed lncRNAs showed that these were broadly similar to that observed in humans. Thus, these were found to be composed of ~30% repeat elements (Figure 5D). Functional analysis identified 540 genes located within 1 Mb of the differentially expressed lncRNAs and showed that these were associated with immune activation and response (Figure 5E), while the fold change in expression of the nearest mRNA was showed to correlate with the changes in AS (*r* = 0.7952, *p* < 0.0001) and lincRNAs (*r* = 0.4209, *p* < 0.0001) (Figure 5F).

Our analysis of sequencing data from multiple mouse cell types identified 210 lncRNAs that were differentially expressed following induction of the innate immune response. These demonstrated comparable characteristics to those observed in humans including cell-specific expression, large regions of repeat sequences, and correlation between their expression and that of local inflammatory genes.

### Identification of Microdomains in Differentially Expressed Human and Mouse lncRNAs

**Figure 6:**
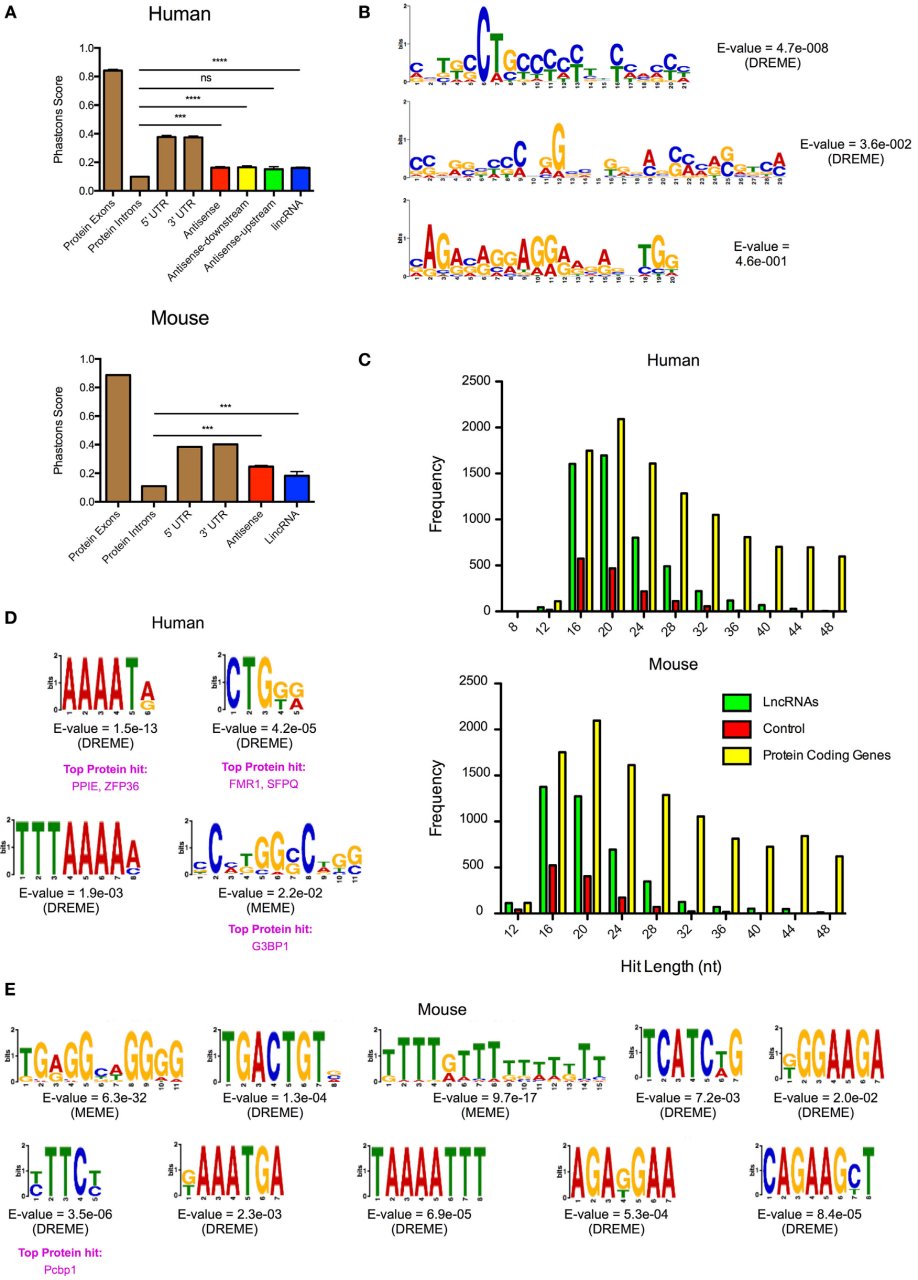
Identification of conserved microdomains in the differentially expressed human and mouse long non-coding RNAs (lncRNAs). **(A)** PhastCons analysis of the conservation of the differentially expressed lncRNA species in human and mouse cells compared with the exon, intronic, and untranslated regions (UTRs) of protein-coding genes. **(B)** Identification of conserved microdomains within human lncRNA sequences using the MEME-suite (following removal of repeat sequences). **(C)** Distribution of hits obtained from the BLASTn analysis of lncRNAs, matched random controls, and matched protein-coding genes in human and mouse. **(D,E)** Identification of conserved microdomains within human **(D)** and mouse **(E)** BLASTn data.

It has been speculated that the action of lncRNAs is mediated through microdomains that interact with proteins or undergo base pairing with RNA and/or DNA. To identify potential microdomains, we searched for conserved sequences within our catalogs of differentially expressed lncRNA genes. As previously reported (46), our initial analysis of the evolutionary conservation of lncRNAs showed that these were poorly conserved (Figure 6A). Thus, using PhastCons (seven-way vertebrate), which determines conservation on a 0–1 scale (1 being the most conserved), we obtained values of 0.162, 0.165, 0.151, and 0.161 with the human AS, AS-downstream, AS-upstream, and lincRNAs, respectively. This value was significantly greater than the 0.099 ± 0.002 for the intronic regions of protein-coding genes (*p* < 0.0001—Mann–Whitney *U*-test) but considerably less than the value for exonic, 5′- and 3′-UTRs of protein-coding genes at 0.842 ± 0.001, 0.376 ± 0.011, and 0.373 ± 0.001, respectively (Figure 6A). Similarly, PhastCons analysis of the mouse catalog (vertebrate 60-way) produced values of 0.246 for AS and 0.182 for lincRNAs, which were significantly greater than those seen for intronic regions (*p* < 0.0001—Mann–Whitney *U*-test) but less than the value for exonic, 5′- and 3′-UTRs of protein-coding genes (Figure 6A).

Despite this poor overall evolutionary conservation, we proceeded to look for the presence of microdomains through comparison of the lncRNAs. This was performed following the removal of the repeat sequences using Repeatmasker (see text footnote 5). These lncRNAs gave a mean length of 4.7 kb (human) and 4.0 kb (mouse) and were shown to be rich in AT residues (58% for human and 54% for mouse). Analysis using MEME-ChIP (37) identified three conserved microdomains in the human lncRNA catalog but found nothing within the mouse lncRNA catalog (Figure 6B). In subsequent studies, we employed BLASTn (38) to identify shared regions within the human and mouse lncRNA catalogs. The output was compared with a comparable number of randomly generated control sequences and protein-coding genes, of similar lengths and AT composition. This analysis showed <1% overall conservation but identified 5,130 and 4,199 significant hits of lengths 12–50 nt in the human and mouse lncRNA catalogs, respectively (Figure 6C). This was significantly higher [*p* < 0.0001: one-way analysis of variance (ANOVA)] than the 1,511 and 1,264 regions identified in the human and mouse control sequences. Protein-coding genes showed comparable number of hits to lncRNAs around the peak of 20 nt, but overall number of hits was elevated throughout the 12–50 nt range (Figure 6C). Submission of the BLASTn hits from the human and mouse lncRNA catalogs into MEME-ChIP identified 4 and 10 microdomains, respectively. No microdomains were detected in the controls. When we compared these motifs with the ATtRACT database of RNA-biding proteins and associated motifs (47), we found that a four of them (three in human and one in mouse) had positive hits with known RNA-binding proteins (Figures 6D,E). In general, these proteins were found to be involved in mRNA splicing, stability, and transport. Thus, despite the poor evolutionary conservation, this analysis indicated that the differentially lncRNA contains short conserved regions or microdomains that might be important in mediating their functions and mechanism of action.

### Identification of Syntenic lncRNAs in the Human and Mouse Innate Immune Responses

No homology was observed between the differentially expressed human and mouse lncRNA catalogs. Since it has been suggested that genomic position might be important to their biological action, we compared the human and mouse catalogs to identify those demonstrating synteny. Our analysis showed that 21 (10%) of the differentially expressed human lncRNAs had syntenic versions in mice and included the two human lncRNAs that were differentially expressed in all human cell types, *hXLOC_405581* (which mapped to mouse *mXLOC_025443*) and *hXLOC_367599* (which mapped to *mXLOC_003168*) (Table S10 in Supplementary Material). However, only five of these syntenic mouse lncRNAs were also significantly differentially expressed (*p* < 0.05) in a least one mouse cell type and included *mXLOC_014053* (*hXLOC_246791*), *mXLOC_039871* (*hXLOC_039871*), *ENSMUSG00000097180* (*hXLOC_376116*), *mXLOC_025443* (*hXLOC_405581*), and *mXLOC_044198* (*hXLOC_455493*). With a mean of 9%, BLASTn analysis showed increased conservation across these syntenic genes, compared to all differentially expressed lncRNAs (<1%), although there was a wide variation (0–53%) (Table S10 in Supplementary Material).

### IL7-AS Regulates the Inflammatory Response in Human and Mice

**Figure 7:**
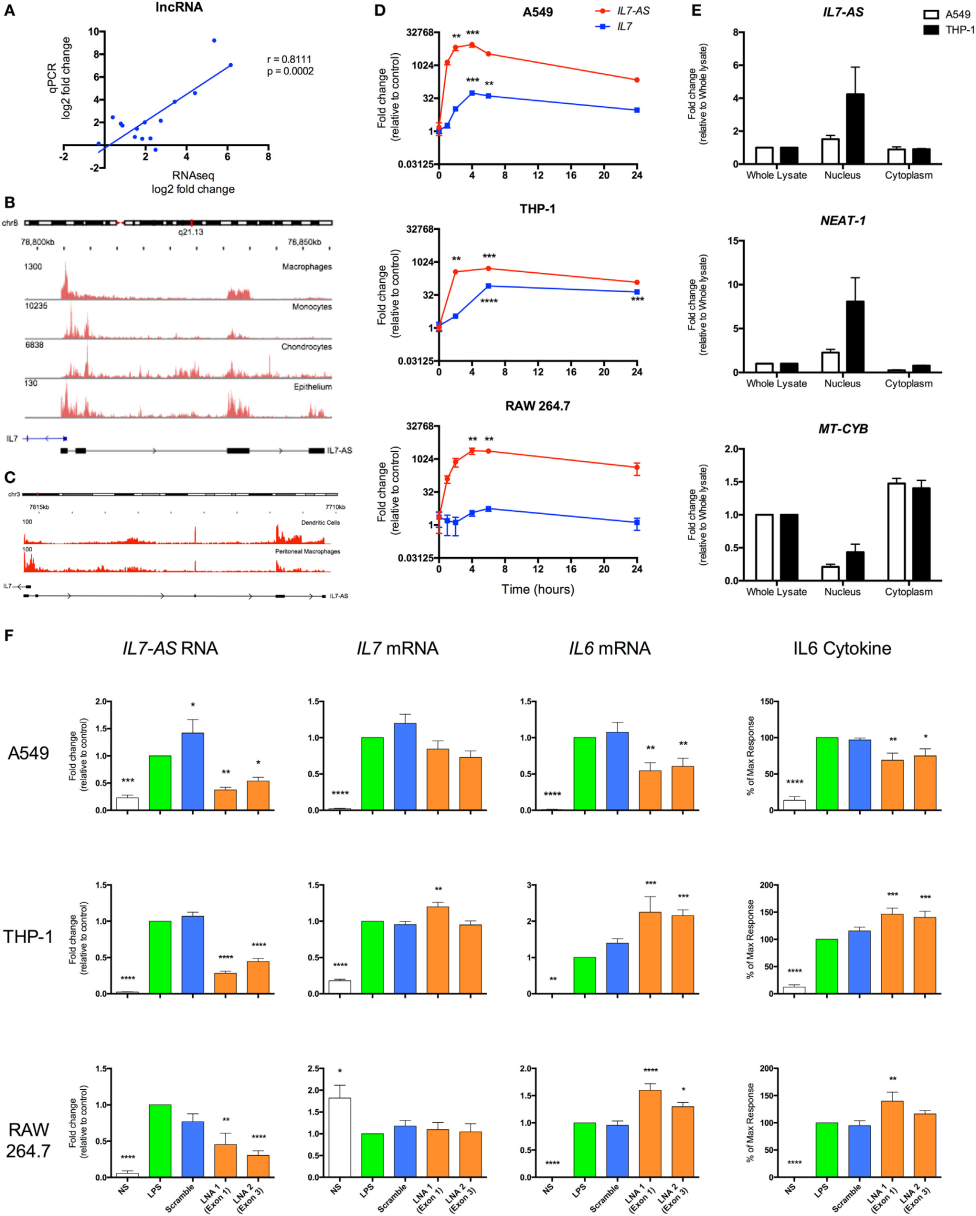
*IL7-AS* regulates the expression and release of IL6 from interleukin-1β (IL1β)- and lipopolysaccharides (LPS)-stimulated human and mouse cells. **(A)** Comparison of fold change in expression of differentially expressed lncRNAs using RNA sequencing and qRT-PCR. Structure and profile of *IL7-AS* expression in **(B)** human and **(C)** mouse cells visualized using the Integrated Genomics Viewer (IGV). **(D)** Time course of *IL7-AS* and *IL7* mRNA production in IL1β-stimulated human alveolar A549 epithelium and LPS-stimulated human THP1 monocytes and mouse RAW 264.7 macrophages (*n* = 3 independent experiments). **(E)** Subcellular distribution of *IL7-AS* in IL1β-stimulated A549 epithelium and LPS-stimulated THP1 monocytes in which *NEAT-1* and mitochondrial-cytochrome b (*MT-CYB*) are employed as markers of nuclear and cytoplasmic fractions, respectively (*n* = 4 independent experiments). **(F)** Effect of transfection with a negative control LNA (scramble) or two antisense LNA (LNA 1 or 2) targeting, respectively, exons 1 and 4 (human cells) or exons 1 and 3 (mouse cells) of *IL7-AS* at a final concentration of 30 nM. Cells were then treated with either IL1β (30 ng/ml) or LPS (1 µg/ml), or left untreated for 24 h, prior to measurement of levels of the stated gene (by q-PCR) or proteins (by ELISA) (*n* = 7–8 independent experiments). Statistical significance was performed using either two-way analysis of variance (ANOVA) for time courses or repeated measure one-way ANOVA with both a Dunnett’s post-test correction, where *p < 0.05, **p < 0.01, ***p < 0.001, and ****p < 0.0001 versus control.

In order to validate the sequencing data, we employed qRT-PCR to measure the levels of five lncRNAs across three human cell types (monocytes, macrophages, and epithelial A549 cells) and showed a significant correlation between the fold changes following differential expression (Figure 7A). To assess the biological relevance of these lncRNA catalogs, we examined the function of the syntenic lncRNAs, *hXLOC_405581* and *mXLOC_025443*, that is differentially expressed in multiple human and mouse cell types. These were renamed *hIL7-AS* and *mIL7-AS* as a result of their AS overlap with the promoter region of *IL7*, a cytokine that has been implicated in T- and B-cell development (48). Analysis of the structure of human and mouse *IL7-AS* showed that these were complex genes that could be assembled potentially into multiple transcripts. In the case of the human *hIL7-AS*, sequencing data indicated the existence of up to nine exons (however, for simplicity, we have only shown the four most represented exons, Figure 7B), which could be assembled into potentially four transcripts of a gene of up to 10,280 nt in length (including 34% repeat sequences). In contrast, the mouse *mIL7-AS* was somewhat less complex containing up to five exons that could be assembled into four potential transcripts giving a gene of an approximate length of 5,043 nt (including 63% repeat sequences, Figure 7C).

To facilitate the functional analysis of *IL7-AS*, experiments were performed using IL1β-stimulated human A549 lung epithelial cells, LPS-stimulated human monocytic THP-1 cells, and LPS-stimulated mouse RAW 264.7 macrophages, which are amenable to transfection. In all cell types, measurement of time courses shows a similar rapid increase in *hIL7-AS* and *mIL7-AS* expressions, peaking between 4 and 6 h and remaining elevated at 24 h (Figure 7D). Examination of *IL7* expression showed a parallel production of *IL7* mRNA, albeit a smaller fold increase, in activated human A549 epithelium and THP-1 monocyte cells, but not mouse RAW 264.7 cells, where *IL7* expression does not seem to be affected by LPS stimulation (Figure 7D). Although *IL7* expression seems to correlate with the expression of *hIL7-AS* in A549 and THP-1 cells, it is interesting to note that the absolute expression of *IL7*, according to our sequencing data, is at least 10 times lower than the expression of *hIL7-AS* (*IL7* = 0.6 FPKM vs *hIL7-AS* = 7.0 FPKM in A549 and *IL7* = 5.5 FPKM vs *hIL7-AS* = 182.2 FPKM in monocyte).

Most lncRNAs present a bias toward nuclear localization, where previous studies of functional lncRNAs have been shown to regulate the transcription of protein-coding genes (7, 49, 50). We therefore investigated the subcellular localization of *IL7-AS* in human cell lines (Figure 7E). Indeed, the expression of *hIL7-AS* was enriched in the nuclear fraction, compared to the whole lysate. We also looked at the expression of *NEAT-1*, a lncRNA known to be mainly located in the nucleus (51) and the mitochondrially encoded cytochrome B (*MT-CYB*) gene, produced from mitochondrial DNA in the cytoplasm (52). As expected, both *NEAT-1* and *MT-CYB* were shown to be enriched, respectively, in the nucleus and in the cytoplasm fraction (Figure 7E), confirming that the separation procedure was successful.

To examine whether *IL7-AS* has a role in the innate immune response, we used AS locked nucleic acid (LNA) to knockdown the expression of *IL7-AS* RNA in both human and mouse. In human, we selected two AS LNAs targeting exon 1 (LNA 1) and exon 4 (LNA 2) that attenuated both IL1β- and LPS-induced *hIL7-AS* production by 50–85% at 24 h (Figure 7F) of A549 and THP-1 cells, respectively. Likewise, in mouse, selected AS LNAs, targeting exon 1 (LNA 1) and exon 3 (LNA 2), showed similar knockdown of *mIL7-AS* than in human cell lines (Figure 7F). Knockdown did not significantly impact upon *IL7* mRNA production indicating that although *IL7-AS* and *IL7* overlap at their promoter region, the action of *IL7-AS* is not mediated through *IL7* regulation *in cis* (Figure 7F). Instead, these results suggest that any potential biological actions of *IL7-AS* might be mediated *in trans*. Indeed, *IL7-AS* knockdown showed significant modulation of the IL1β- and LPS-induced expressions of the pro-inflammatory mediator IL6 on both mRNA production and release of the cytokine in human and in mouse (Figure 7F). Interestingly, IL1β-induced IL6 production was significantly down-regulated in A549 cells upon *IL7-AS* knockdown, while in THP-1 and RAW 264.7 cells, knockdown showed an upregulation of LPS-induced IL6 production (Figure 7F). These results suggest that *IL7-AS* function on IL6 production is cell and/or stimuli specific.

Overall, these studies provide evidence of the utility of using differential expression as the basis for identifying functional lncRNA in the innate immune response and have for the first time identified a lncRNA (i.e., *IL7-AS*) that regulates the inflammatory response in both human and mouse models.

## Discussion

Using next-generation sequencing data from four human and four mouse cell types, we have undertaken the first comprehensive analysis of the changes in lncRNA expression associated with the activation of the innate immune response. This is important since differential expression has commonly provided the initial step in the search for functional lncRNAs and has led to the identification a number that regulate the associated inflammatory response including *PACER* (13), *THRIL* (14), *lnc-IL7R* (15), and *IL1β-RBT46* (16) in humans and *lincRNA-COX2* (17, 18), *lincRNA-EPS* (19), and *lincRNA-Tnfaip3* (20) in mice. Differential expression has also been employed to compare T- and B-cell populations and identified lncRNAs that regulate multiple aspects of the adaptive immune response including activation, proliferation, and differentiation (53–55). Using this approach, we have demonstrated differential expression of 204 human and 210 mouse lncRNAs, which included *PACER* (13), *IL1β-RBT46* (16), *lincRNA-COX2* (17, 18), and *lincRNA-EPS* (19). Intriguingly, we were unable to detect the expression of *THRIL* (14), *lnc-IL7R* (15), or *lincRNA-Tnfaip3* (20), which were all located within the 3′ UTRs of known protein-coding genes and were initially detected using microarrays. In future, we therefore believe that visual annotation of sequencing data should be the method of choice when identifying novel lncRNAs. In order to produce as comprehensive a lncRNA catalog as possible, sequencing of two of the eight cell types (human epithelial A549 cells and the mouse macrophage RAW264.7 cells) was undertaken after polyA+ selection (rather than ribozero selection). This is likely to influence the comparison between cell types and, specifically, might reduce the number of lncRNAs detected, since we would be unable to identify those lacking polyA+ tails. Although it is difficult to assess the potential impact of polyA+ versus ribozero isolation, the similarity in the numbers of differentially expressed lncRNAs in epithelial A549 cells (39 lncRNAs), compared with monocytes (105 lncRNAs), macrophages (50 lncRNAs), and chondrocytes (65 lncRNAs), indicates that we may be omitting only a small number.

As a part of our analysis, we also investigated the changes in single exon transcripts, which are traditionally excluded from the lncRNA classification (which requires two or more exons). This work suggests that, although a number of these are likely to regulate the innate immune response (i.e., *PACER*) (13), the large numbers and the wide variation between cell types indicate that the majority represent transcriptional noise and/or are an artifact of the analysis pathway. This conclusion is supported by the ChIPseq analysis in human monocytes that showed a poor overlap between markers of active transcription and enhancers/promoters.

Analysis of the full-length genes demonstrated weak conservation through evolution, as well as between the lncRNAs (<1%) and showed no homology between the human and mouse catalogs. However, a combination of BLASTn and MEME-ChIP allowed the identification of multiple conserved microdomains of lengths 5–30 nt. We speculate that these microdomains might mediate the actions of lncRNAs, either through protein binding and/or base pairing to RNA/DNA. Previous reports have uncovered only lncRNA–protein interactions, including an action of *lincRNA-COX2* (17, 18), *THRIL* (14), and *lincRNA-EPS* (19) through heterogeneous ribonucleoproteins and *PACER via* the p50 component of NF-κB (13). Of relevance, the identification of microdomains was performed following the removal of the repeats that comprised ~30% of lncRNA sequences. However, it is possible that repeats are important in mediating the action of lncRNA, with previous studies showing that *Alu* repeats can activate the inflammasome (51) and contain binding sites for transcription factors involved in regulating the macrophage response to *Mycobacterium tuberculosis* infection (56).

It has also been suggested that the maintenance of genomic position relative to protein-coding genes (synteny) might be important in determining the lncRNA function. Comparison across humans and mouse identified 22 syntenic lncRNAs, of which five were differentially expressed in both species. This included *IL7-AS* (located AS to *IL7*), which was induced across multiple human and mouse cell types and demonstrated the largest changes in absolute expression among the syntenic lncRNAs. Measurement of *IL6* transcription and secretion showed that *IL7-AS* was a positive regulator of IL1β-induced inflammatory response in human A549 epithelial cell but a negative regulator in LPS-stimulated human THP-1 monocytes and mouse RAW 264.7 macrophages. Given our previous report showing that *IL7-AS* (or *CILinc02*) was a negative regulator of IL1β-stimulated IL6 release from human chondrocytes (57), this indicates that its actions are cell-type specific rather than stimulus specific. Future studies will need to ascertain whether this is related to alternative splicing and/or cell-specific differences in lncRNA mechanisms. In addition, since *IL7-AS* is the first lncRNA to demonstrate function in the innate immune response in both human and mouse cell models, this provide an opportunity to compare the physiological role of lncRNAs across these two species.

In summary, we have for the first time cataloged and characterized those lncRNAs that are differentially expressed in multiple human and mouse cell types following activation of the innate immune response. However, further studies will be necessary to determine which other lncRNAs are functional from those two catalogs. Indeed, a refined list of functional lncRNAs could give us fewer and/or more defined microdomains. It is envisaged that this will provide an important resource for the discovery of functional lncRNAs and elucidation of their mechanism of action.

## Author Contributions

BR undertook the majority of laboratory based studies, assisted in the experimental design and analysis of data, and contributed to the writing of the manuscript. JH contributed toward the laboratory based studies, the bioinformatics analysis, and the writing of the paper. LD assisted with experimental design. SJ assisted with experimental design and contributed to the writing of the paper. ML conceived of the experimental design, undertook the majority of the analysis of data, and contributed to the writing of the manuscript.

## Conflict of Interest Statement

The authors declare that the research was conducted in the absence of any commercial or financial relationships that could be construed as a potential conflict of interest.

## Footnotes

^1^http://www.bioinformatics.babraham.ac.uk/projects/fastqc.

^2^https://www.ncbi.nlm.nih.gov/sra.

^3^http://cpc.cbi.pku.edu.cn.

^4^https://genome.ucsc.edu/cgi-bin/hgTables.

^5^http://www.repeatmasker.org.

^6^http://rsat.sb-roscoff.fr.

^7^http://www.ensembl.org/index.html.

^8^http://genome.ucsc.edu/cgi-bin/hgLiftOver.

^9^http://bejerano.stanford.edu/great/public.

## Supplementary Material

Data Sheet S1 Principle component analysis and hierarchical clustering. Analysis of the distribution of samples using principle component analysis and hierarchical clustering in monocytes, macrophages, epithelium, and chondrocytes.

Data Sheet S2 Expression of antisense, lincRNA, and antisense-upstream/downstream are not correlated with the nearest protein-coding gene. The correlation (Spearman’s correlation) between the expression of antisense **(A)**, antisense upstream and downstream **(B)**, and lincRNAs **(C)**, demonstrating an FPKM > 1 in non-stimulated cells was examined using data from all four cell types (monocytes, macrophages, epithelium, and chondrocytes).

Data Sheet S3 Expression of *THRIL* in human monocytes and macrophages. Profile of forward (red) and reverse (blue) sequence reads in control and LPS-stimulated human monocytes and macrophages indicates the presence of *BRI3BP* but not the *THRIL*.

Table S1 LNA antisense sequence and qRT-PCR probes.

Table S2 Bioinformatics software and websites.

Table S3 Gencode v23 protein-coding genes across four human cell types.

Table S4 Long non-coding RNA expression across four human cell types.

Table S5 Differentially expressed long non-coding RNA expression across four human cell types.

Table S6 Differentially expressed single exon long non-coding RNA expression across four human cell types.

Table S7 Mouse Gencode m12 gene expression across four mouse cell types.

Table S8 Long non-coding RNA expression across four mouse cell types.

Table S9 Differentially expressed long non-coding RNA expression across four mouse cell types.

Table S10 Syntenic long non-coding RNAs between humans and mouse.

## Abbreviations

BMDM, bone marrow-derived macrophages; mRNA, messenger RNA; ncRNA, non-coding RNA; lncRNA, long non-coding RNA; lincRNA, long intergenic non-coding RNA; IL, interleukin; LPS, lipopolysaccharide; nt, nucleotide.
