# Supplementary figures and images for: Catalog of Differentially Expressed Long Non-Coding RNA following Activation of Human and Mouse Innate Immune Response
Source: Front Immunol. 2017 Aug 29;8:1038. doi: 10.3389/fimmu.2017.01038 (PMC5581803; doi:10.3389/fimmu.2017.01038)

Monocytes

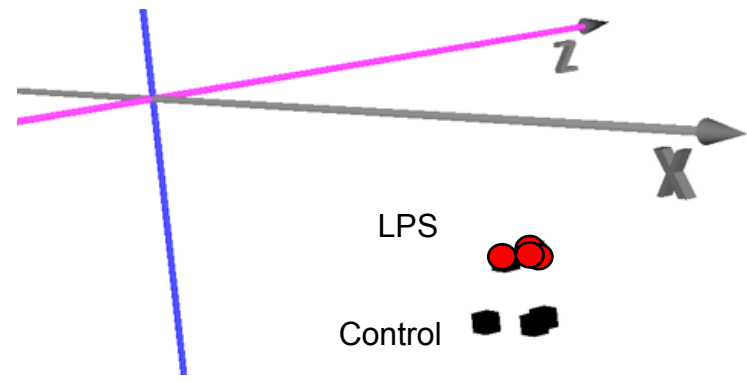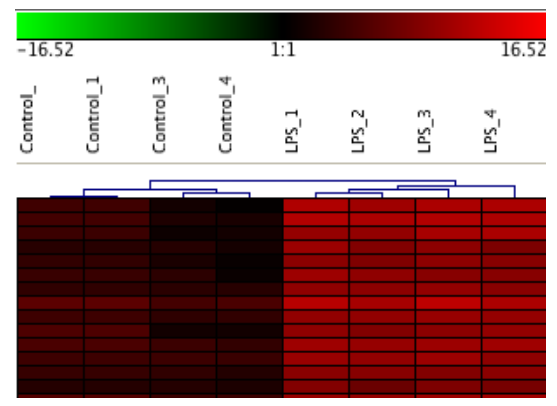

Macrophages

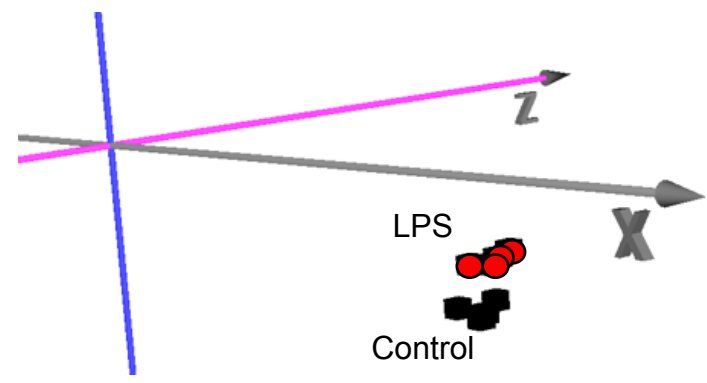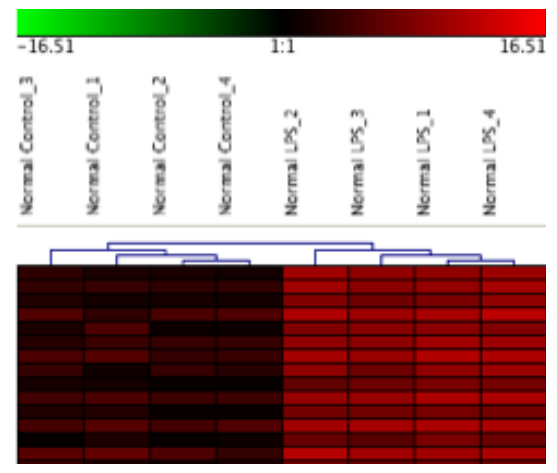

Epithelium

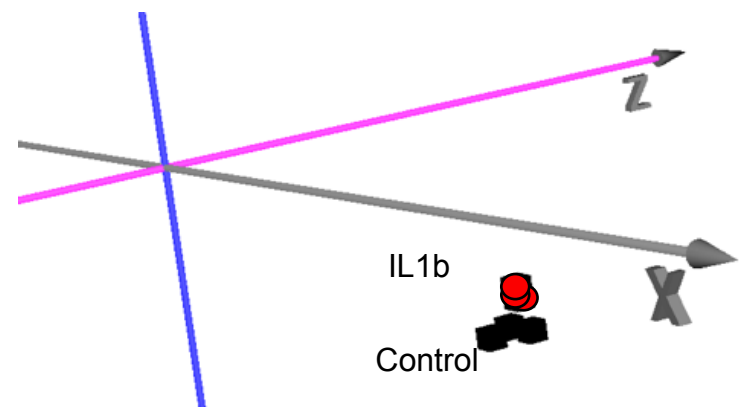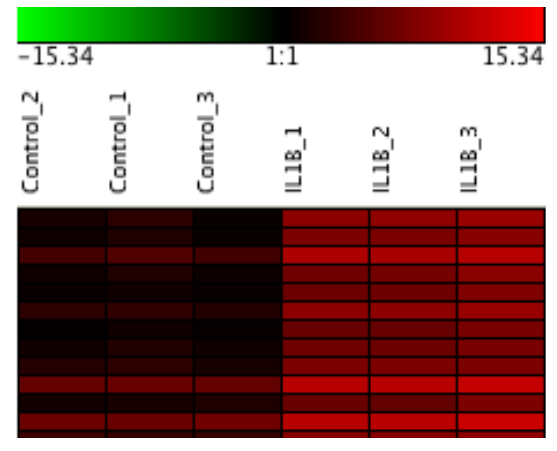

Chondrocytes

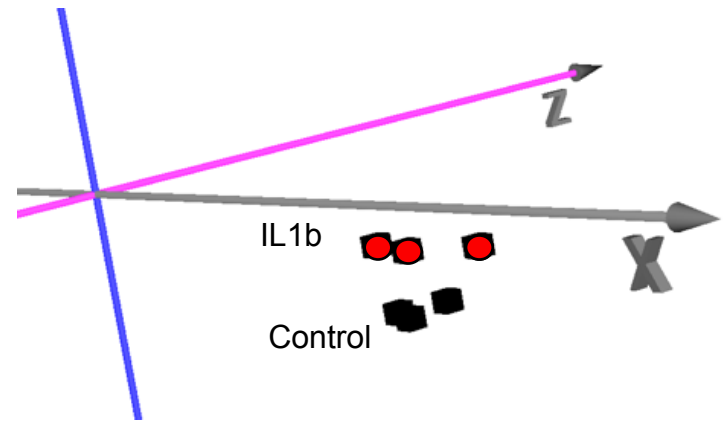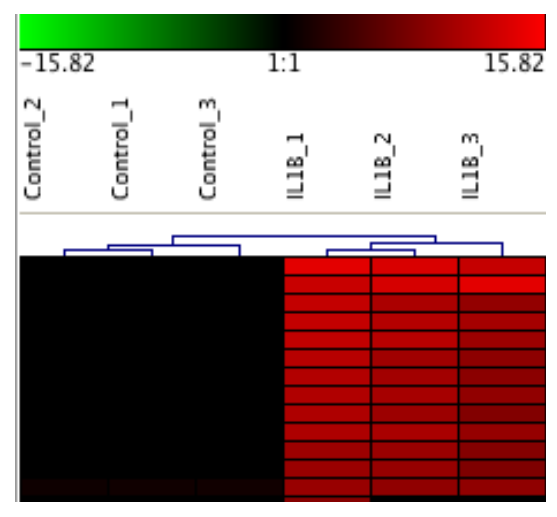

Supplement: Data Sheet S1 — Principle component analysis and hierarchical clustering. Analysis of the distribution of samples using principle component analysis and hierarchical clustering in monocytes, macrophages, epithelium, and chondrocytes. [file data_sheet_1.pdf]

(A)

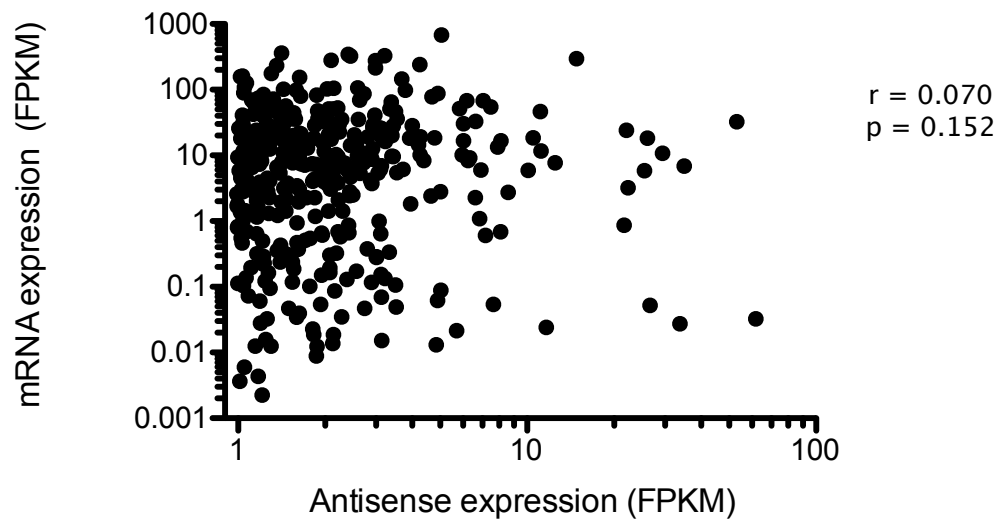

(B)

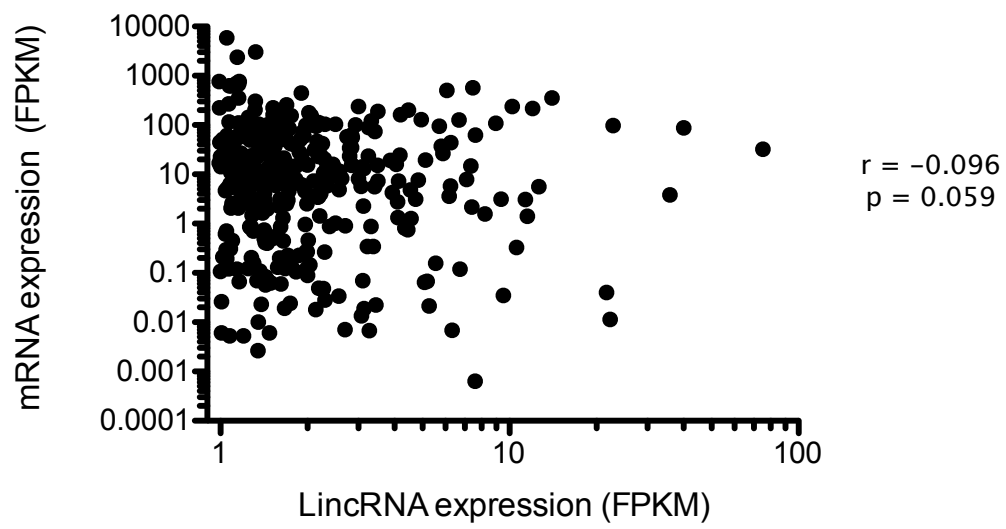

(C)

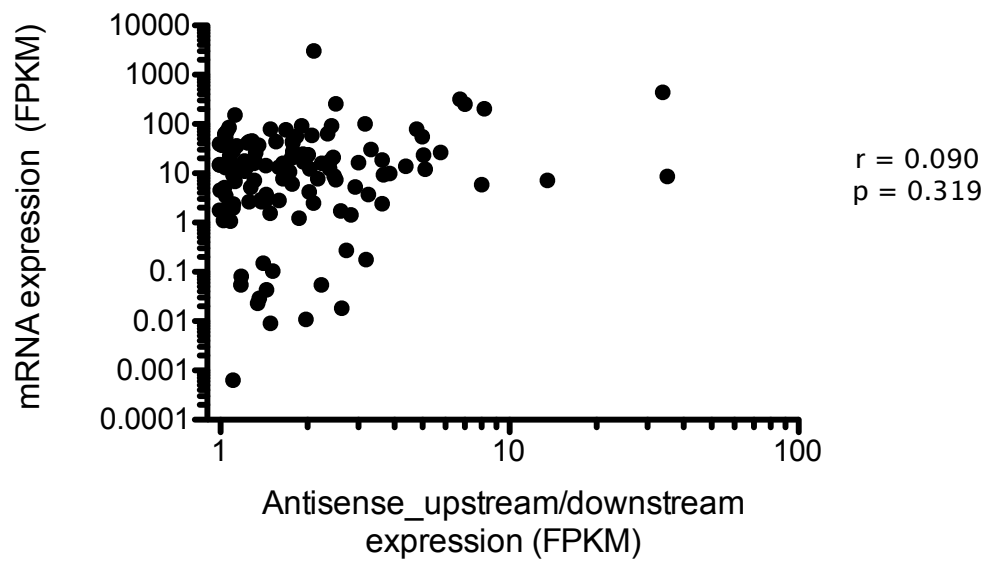

Supplement: Data Sheet S2 — Expression of antisense, lincRNA, and antisense-upstream/downstream are not correlated with the nearest protein-coding gene. The correlation (Spearman’s correlation) between the expression of antisense (A), antisense upstream and downstream (B), and lincRNAs (C), demonstrating an FPKM > 1 in non-stimulated cells was examined using data from all four cell types (monocytes, macrophages, epithelium, and chondrocytes). [file data_sheet_2.pdf]

chr12

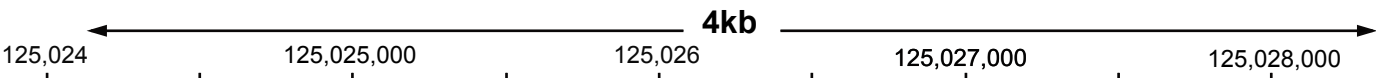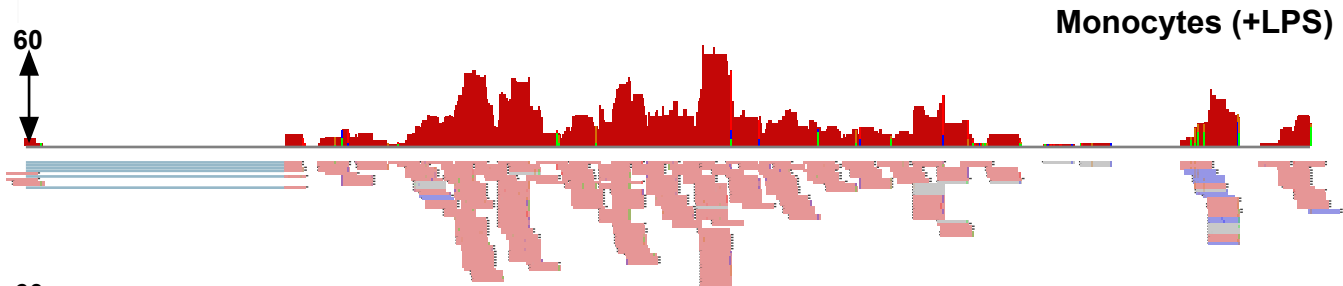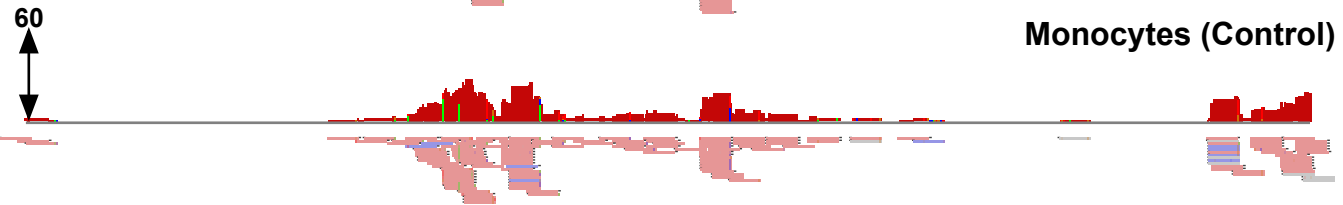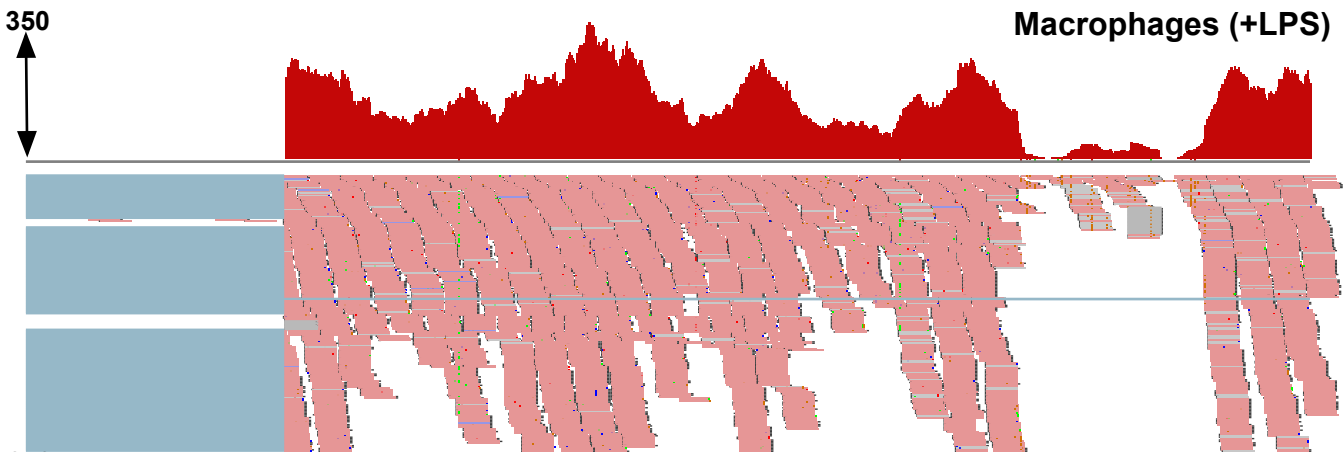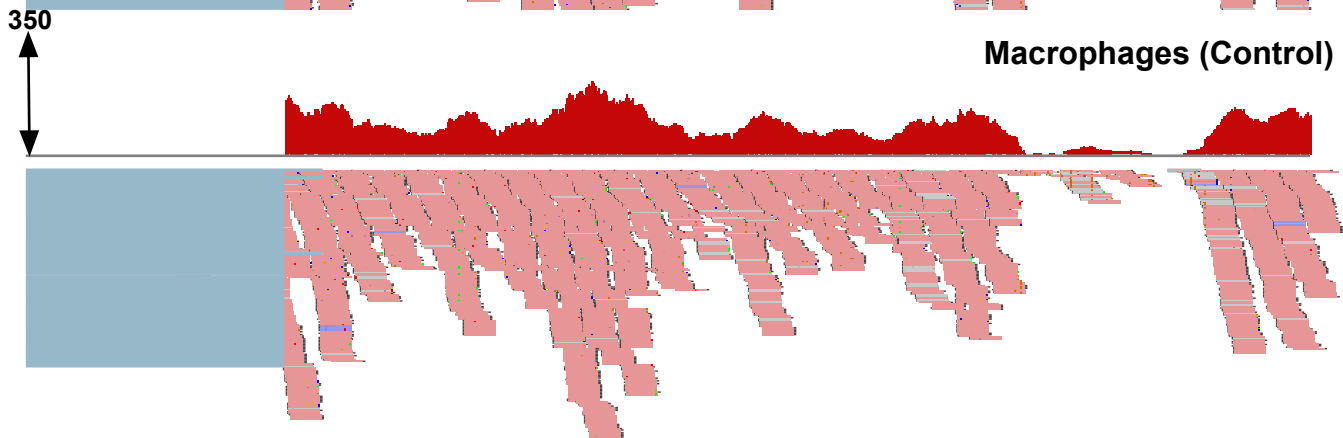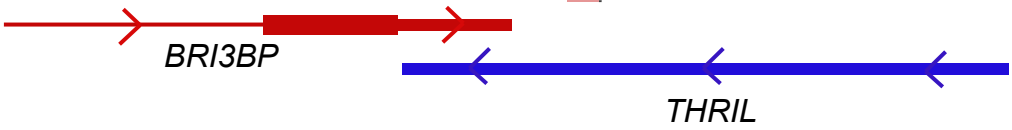

Supplement: Data Sheet S3 — Expression of THRIL in human monocytes and macrophages. Profile of forward (red) and reverse (blue) sequence reads in control and LPS-stimulated human monocytes and macrophages indicates the presence of BRI3BP but not the THRIL. [file data_sheet_3.pdf]
