# Supplementary material for: Catalog of Differentially Expressed Long Non-Coding RNA following Activation of Human and Mouse Innate Immune Response
Source: Front Immunol. 2017 Aug 29;8:1038. doi: 10.3389/fimmu.2017.01038 (PMC5581803; doi:10.3389/fimmu.2017.01038)
Supplement: Table S2 — Bioinformatics software and websites. [file table_2.docx]

**Software and websites**

| ATtRACT | Girolamo et al. 2016 (47) | https://attract.cnic.es/ |
| --- | --- | --- |
| Bamtools | Barnett et al., 2011 (25) | https://github.com/pezmaster31/bamtools |
| Bedtools | Quinlan and Hall, 2010 (30) | http://bedtools.readthedocs.io/en/latest/ |
| Blast+ | Camancho et al 2009 (38) | ftp://ftp.ncbi.nlm.nih.gov/blast/executables/blast+/LATEST/ |
| Bowtie 2 | Langmead and Salzberg, 2012 (41) | http://bowtie-bio.sourceforge.net/bowtie2/index.shtml |
| Coding Potential calculator | Kong et al., 2007 (31) | http://cpc.cbi.pku.edu.cn |
| Cufflinks/CuffNorm/  Cuffmerge/CuffDiff | Trapnell et al., 2010 (28) | https://github.com/cole-trapnell-lab/cufflinks |
| deepTools | Ramirez et al., 2016 (42) | http://deeptools.readthedocs.io/en/latest/content/installation.html |
| FASTQC | Andrews S, 2010 | http://www.bioinformatics.babraham.ac.uk/projects/fastqc |
| Galaxy | Afgan et al., 2016 (35) | https://usegalaxy.org |
| Genesis | Strun et al., 2002 (33) | http://genome.tugraz.at/genesisclient/genesisclient_description.shtml |
| HISAT2 | Kim et al., 2015 (23) | https://ccb.jhu.edu/software/hisat2/index.shtml |
| MACS2 | Zhang et al., 2008 (43) | https://github.com/taoliu/MACS |
| Meme-Suite | Bailey et al., 2009 (37) | http://meme-suite.org |
| Repeatmasker | Smit AFA, Hubley R & Green P, 2013-15 | http://www.repeatmasker.org |
| RSAT | Medina-Rivera et al 2015 (39) | http://www.rsat.eu |
| Samtools | Li et al., 2009 (24) | http://samtools.sourceforge.net |
| SRA |  | https://trace.ncbi.nlm.nih.gov/Traces/sra/sra.cgi?view=software |
| Stringtie | Pertea et al., (2015) (26) | https://ccb.jhu.edu/software/stringtie/#install |
| Tophat2 | Kim et al., 2013 (22) | https://ccb.jhu.edu/software/tophat/index.shtml |
| UCSC, genome browser | Kent et al. 2002 (36) | https://genome.ucsc.edu |
